# Discovery and Evaluation of Novel Calenduloside E Derivatives Targeting HSP90β in Ox-LDL-Induced HUVECs Injury

**DOI:** 10.3390/ph19010090

**Published:** 2026-01-02

**Authors:** Fang Han, Huiqi Fang, Guangyu Li, Di Deng, Guibo Sun, Yu Tian

**Affiliations:** State Key Laboratory for Quality Ensurance and Sustainable Use of Dao-di Herbs, Institute of Medicinal Plant Development, Chinese Academy of Medical Sciences and Peking Union Medical College, Beijing 100193, China

**Keywords:** atherosclerosis, Calenduloside E, heat shock protein 90β, ticagrelor, HUVECs

## Abstract

**Background**: Atherosclerosis (AS) serves as the primary pathological basis for cardiovascular disease-related deaths worldwide, posing a severe threat to public health security. Heat shock protein 90 (HSP90) plays a crucial regulatory role in the pathological progression of AS, emerging as a potential target for anti-atherosclerosis drug development in recent years. Calenduloside E (CE) is a pentacyclic triterpenoid saponin isolated from *Aralia elata* (Miq.) Seem. Previous studies have confirmed its anti-atherosclerotic activity, but its weak efficacy and narrow therapeutic index limit its clinical application. In this study, the CE scaffold was hybridized with a ticagrelor-derived fragment to enhance anti-atherosclerotic activity. In this study, the CE scaffold was hybridized with a ticagrelor fragment to achieve improved activity. **Methods**: Based on the principle of molecular hybridization, CE was linked to the active fragment of ticagrelor via a PEG chain. Ten CE derivatives were synthesized by modifying the sugar substituents. In vitro experiments were conducted to detect cytotoxicity and protective activity against ox-LDL-induced HUVECs injury. Molecular docking and Surface Plasmon Resonance (SPR) assays were used to evaluate the interaction between CE derivatives and the known target HSP90β. Combined with Microscale Thermophoresis (MST), SwissTargetPrediction, and molecular docking, other potential targets of CE derivatives were identified. **Results**: In the ox-LDL-induced HUVECs injury model, all compounds except C2 and C9 exhibited protective activity. Among these compounds, compound **C5** exhibited the optimal protective effect, with an EC_50_ value of 1.44 μM. Molecular docking results revealed that both **C5** and CE could bind to HSP90β by forming hydrogen bonds with the key amino acid Asp93. Additionally, SPR results indicated that **C5** and CE had similar binding affinities to HSP90β, with dissociation constants (K_D_) of 1.73 μM and 1.72 μM, respectively. MST demonstrated that **C5** binds to HSP90β with an affinity 111 times higher than that of ticagrelor. SwissTargetPrediction and molecular docking identified P2Y12 as another potential target of derivative **C5**. **Conclusions**: Compound **C5** exerts protective effect against ox-LDL-induced HUVECs injury by targeting HSP90β. Its effective concentration is significantly improved compared with that of the parent CE, which provides a possibility for reducing clinical dosage and toxic side effects in subsequent studies. Furthermore, **C5** may exert its effects by targeting another potential target, P2Y12, offering references for the rational design of novel anti-atherosclerotic drugs.

## 1. Introduction

Atherosclerosis (AS) is the primary pathological basis for cardiovascular disease-related deaths worldwide [1]. Its core features include subendothelial lipid deposition, inflammatory cell infiltration, and fibrous cap formation [2]. These changes gradually lead to vascular lumen stenosis, decreased elasticity, and increased risk of plaque rupture [3,4,5]. AS is not an isolated vascular lesion; instead, it is a multifactorial pathological process involving endothelial dysfunction [6], abnormal proliferation and migration of vascular smooth muscle cells (VSMCs) [7], platelet activation and aggregation [8], and chronic inflammatory responses [9]. Epidemiological data show that approximately 17.9 million people worldwide die from AS-related cardiovascular diseases each year [10]. Moreover, the age of onset is trending younger. AS has thus become a major disease that seriously threatens public health security. Currently, commonly used clinical intervention drugs, such as statins [11] and antiplatelet agents [12], mostly target a single pathological link. These drugs are limited in their efficacy and are associated with adverse reactions such as bleeding and liver injury [13,14,15]. Therefore, there is an urgent clinical need to develop new anti-atherosclerosis drugs.

Heat shock protein 90 (HSP90) is a highly conserved molecular chaperone protein. It plays a key regulatory role in the pathological progression of atherosclerosis (AS) and has emerged as a potential target for anti-atherosclerosis drug development in recent years. HSP90 can directly bind to endothelial nitric oxide synthase (eNOS), forming an HSP90-Akt-eNOS complex. This complex prevents eNOS degradation, thereby enhancing nitric oxide (NO) production [16,17]. Consequently, it maintains vascular endothelial barrier function and inhibits endothelial cell apoptosis. Meanwhile, HSP90 can regulate the nuclear factor-κB (NF-κB) signaling pathway. This regulation reduces the secretion of inflammatory factors such as tumor necrosis factor-α (TNF-α) and interleukin-6 (IL-6), alleviating inflammatory infiltration in vascular endothelium [18]. In addition, inhibiting HSP90 can induce G1 phase arrest of VSMCs. This significantly suppresses the proliferation and invasion abilities of VSMCs, further reducing intimal hyperplasia and plaque formation [19]. In terms of regulating the plaque inflammatory microenvironment and maintaining plaque stability, HSP90 is highly expressed in inflammation-enriched areas of AS plaques and thin-cap unstable plaques. This high expression promotes the recruitment of macrophages to the plaque site and the formation of foam cells. In contrast, targeted inhibition of HSP90 can improve plaque phenotype by reducing foam cell apoptosis and inhibiting lipid accumulation [20]. These studies fully confirm that HSP90 regulates multiple pathological links of AS through multiple dimensions, making it an important molecular target for anti-AS drug development.

**Figure 1:**
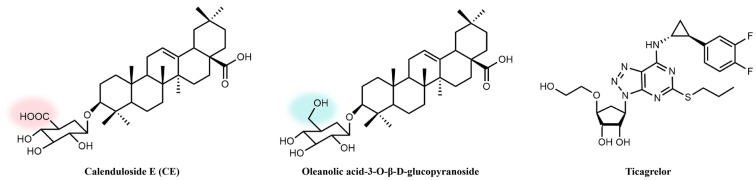
Structures of Calenduloside E, oleanolic acid-3-O-β-D-glucopyranoside and ticagrelor.

Calenduloside E (CE) is a pentacyclic triterpenoid saponin isolated from *Aralia elata* (Miq.) Seem (Figure 1). Previous studies have confirmed its effects in resisting myocardial ischemia–reperfusion injury [21], inhibiting endothelial cell apoptosis, and alleviating oxidative stress. In atherosclerosis-related studies, CE can inhibit calcium overload in cardiomyocytes by regulating the interaction between L-type calcium channels (LTCCs) and BAG3 protein [22]. This further improves vascular endothelium-dependent diastolic function. Meanwhile, CE can target the SIRT2-NLRP3 inflammasome axis. It reduces the acetylation level of NLRP3 at the K24 site, inhibits macrophage pyroptosis, and suppresses the release of pro-inflammatory factors such as IL-1β and IL-6 [23]. These effects alleviate inflammatory infiltration in adipose tissue and vascular walls. Further studies have revealed that the intervention effect of CE on AS also involves the regulation of metabolic reprogramming. In the oxidized low-density lipoprotein (ox-LDL)-induced RAW264.7 macrophage model, CE can upregulate the expression of Kruppel-like factor 2 (KLF2) [24]. This upregulation inhibits the activities of glycolysis key enzymes PFKFB3 and GLUT1 and reduces lactic acid production. Consequently, it suppresses M1 macrophage polarization and decreases foam cell formation. To further clarify the target of CE in exerting anti-atherosclerosis effects, we previously designed and synthesized a click chemistry probe [25,26]. The results showed that CE can exert endothelial cell protection and anti-apoptotic effects by targeting HSP90β.

However, as a natural product, CE has relatively low anti-atherosclerotic activity. Its potential as a single therapeutic drug is limited, thus requiring structural modification strategies to enhance its biological activity. Ticagrelor, a cyclopentyltriazolopyrimidine derivative, is a clinically widely used P2Y12 receptor antagonist. It can directly bind to the P2Y12 receptor without hepatic metabolic activation, rapidly inhibiting platelet activation and aggregation. In the treatment of acute coronary syndrome (ACS), ticagrelor significantly reduces the incidence of endpoint events such as cardiovascular death and myocardial infarction [27,28,29]. Additional studies have demonstrated that ticagrelor can notably reduce the necrotic core area of aortic sinus plaques and increase the fibrous cap thickness in ApoE^−/−^ mice [30]. Nevertheless, ticagrelor is associated with adverse reactions like increased bleeding risk and dyspnea in clinical application, which limits its long-term use [31]. Based on this, we propose the structural hybridization of the key pharmacophore fragment of ticagrelor with CE. The aim is to optimize the molecular structure of CE, enhance its regulatory activity on AS pathological links, and improve its development potential as an anti-atherosclerotic drug.

This study utilized Calenduloside E as the parent core structure and synthesized 10 novel hybrid compounds by conjugating the active pharmacophore of ticagrelor via a PEG chain. Through structural identification of the target compounds and in vitro evaluation of their anti-atherosclerotic activity, the present work systematically investigates their protective effects on vascular endothelium and clarifies the structure–activity relationship. These findings aim to provide experimental data and theoretical support for the development of novel anti-atherosclerotic drugs with high efficacy and low toxicity.

## 2. Results and Discussion

### 2.1. Design and Synthesis of Derivatives

Calenduloside E (CE) is a natural triterpenoid saponin. Its aglycone is oleanolic acid, and the 3-hydroxyl group of this aglycone is linked to D-glucuronic acid. Structurally, CE differs from oleanolic acid-3-O-β-D-glucopyranoside in the group at the C–6 position of the glucopyranose ring. CE has a carboxyl group at this position, while oleanolic acid-3-O-β-D-glucopyranoside has a hydroxyl group. Compared with oleanolic acid-3-O-β-D-glucopyranoside, Calenduloside E is more expensive and has a lower synthetic yield. Therefore, we replaced CE with oleanolic acid-3-O-β-D-glucopyranoside as the parent core structure for further modification. Based on the principle of drug hybridization, its key pharmacophore fragment was linked with that of ticagrelor. This strategy aims to obtain compounds with excellent activity and novel structures, thereby achieving better myocardial protective effects. The design and synthesis of the target compounds generally involve four steps, including modification of the sugar donor, synthesis of oleanolic acid-3-O-β-D-glucopyranoside, amidation at the C28 position of oleanolic acid-3-O-β-D-glucopyranoside, and hybridization with the ticagrelor intermediate.

**Scheme 1:**
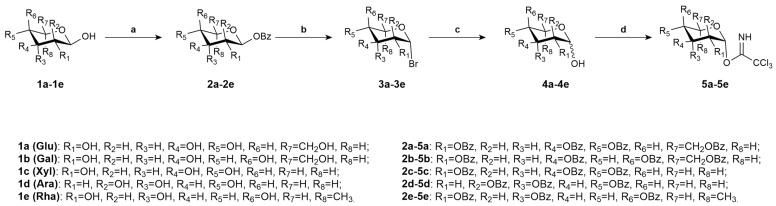
Reagents and conditions: (a) BzCl, pyridine, rt, 18 h; (b) 33% HBr–HOAc, DCM, rt, 5 h; (c) Ag_2_O, acetone, H_2_O, rt, 3 h; (d) CCl_3_CN, K_2_CO_3_, DCM, rt, 6 h.

During the monosaccharide synthesis process, the Schmidt method [32] was selected for glycosylation. This method offers advantages such as easy preparation, good stability, high yield, excellent stereoselectivity, and mild reaction conditions. Glucose (**1a**), galactose (**1b**), xylose (**1c**), arabinose (**1d**), and rhamnose (**1e**) were converted into sugar donors **5a**–**5e,** respectively (Scheme 1).

**Scheme 2:**
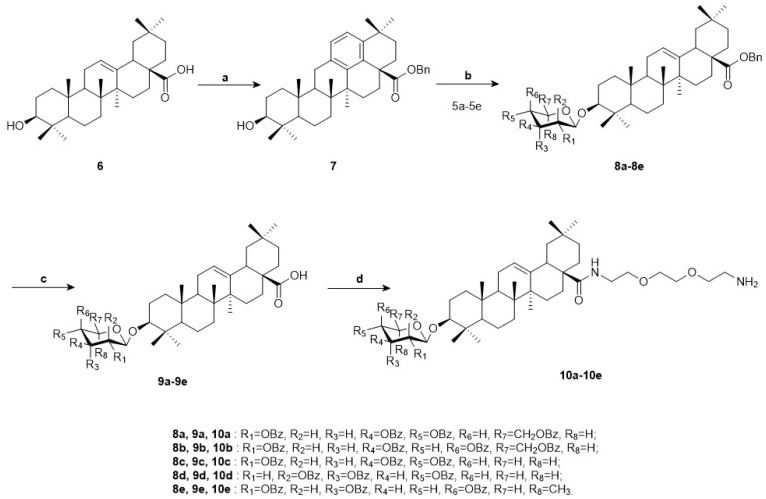
Reagents and conditions: (a) BnBr, K_2_CO_3_, TBAB, DCM, rt, 18 h; (b) **5a**–**5e**, TMSOTf, DCM, rt, 2–4 h; (c) H_2_, Pd–C (10%), EtOAc, reflux, 4–6 h; (d) HOBT, EDCI, 1,8-diamino-3,6-dioxaoctane, rt, 4–16 h.

Meanwhile, oleanolic acid (**6**) and benzyl bromide were dissolved in anhydrous dichloromethane in the presence of potassium carbonate and tetrabutylammonium bromide for benzylation reaction, yielding intermediate **7**. Catalyzed by Lewis acid, intermediate **7** underwent glycosylation reaction with glycosyl donors **5a**–**5e** using trimethylsilyl trifluoromethanesulfonate (TMSOTf), generating compounds **8a**–**8e**. Subsequently, the obtained glycosides (**8a**–**8e**) were hydrogenated under atmospheric pressure using 10% palladium on carbon (Pd/C) as the catalyst, resulting in compounds **9a**–**9e**. The intermediate was dissolved in anhydrous dichloromethane. Condensing agents HOBT and EDCI were added, followed by the addition of 1,8-diamino-3,6-dioxaoctane under ice bath conditions for amidation condensation reaction, which afforded compounds **10a**–**10e** (Scheme 2).

The synthesis of the active fragment of ticagrelor was carried out concurrently. In the design and synthesis process, we retained the core active fragment of ticagrelor, namely the cyclopentyl–triazolopyrimidine (CPTP) scaffold—a fused-ring structure consisting of a cyclopentyl moiety covalently linked to a 1,2,3-triazolo[4,5-d] pyrimidine heterocycle [33,34]. This rigid fused-ring system serves as the fundamental structural basis for ticagrelor’s biological activity: it enables direct, reversible binding to the P2Y12 receptor’ s orthosteric pocket without requiring metabolic activation, and forms key hydrogen bonds and hydrophobic interactions with critical receptor residues (e.g., Arg256 and Tyr259) to stabilize the receptor in an inactive conformation [35].

**Scheme 3:**
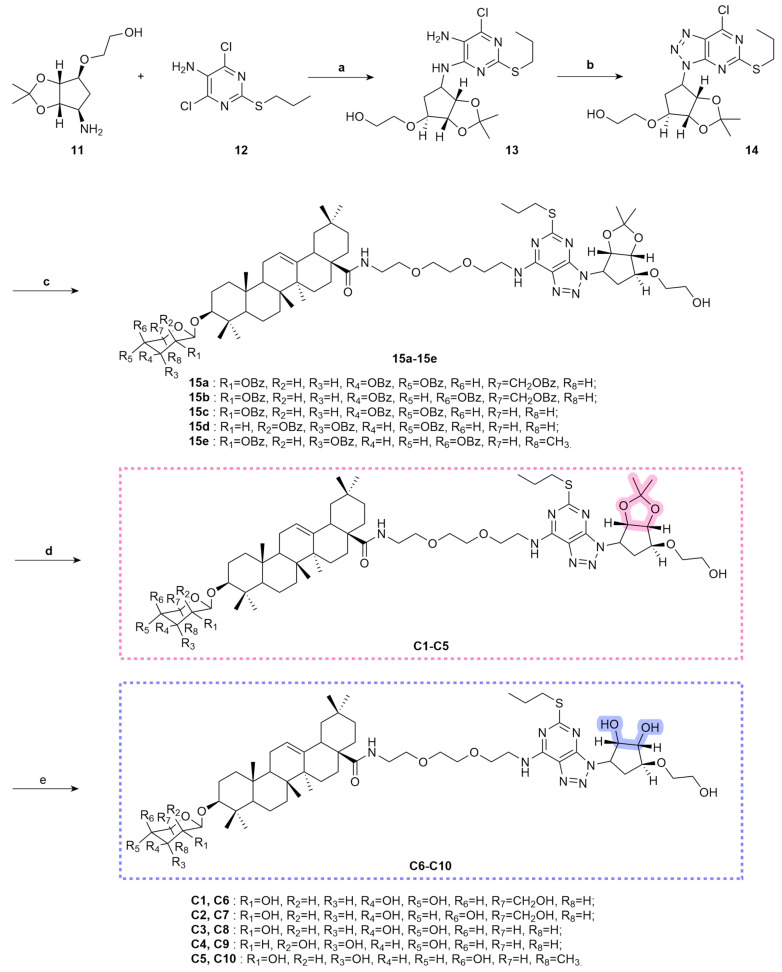
Reagents and conditions: (a) Et_3_N, 1,4-dioxane, 110 °C reflux, 24 h; (b) NaNO_2_, HAc, EA, rt, 8 h; (c) **10a**–**10e**, DIPEA, DCM, rt, 10 h; (d) CH_3_ONa, CH_3_OH, DCM, rt, 6 h; (e) 1N HCl, CH_3_OH, rt, 2 h.

Ticagrelor tartrate (**11**) and 4,6-dichloro-5-amino-2-propylthiopyrimidine (**12**) were dissolved in 1,4-dioxane. Triethylamine was added, and the mixture was heated to reflux at 110 °C to obtain intermediate **13**. Subsequently, intermediate **13** was dissolved in ethyl acetate. Acetic acid was added, followed by the dropwise addition of an aqueous sodium nitrite solution. The reaction was conducted under ice bath conditions to yield intermediate **14**. Compounds **10a**–**10e** and intermediate **14** were dissolved in dichloromethane. N,N-diisopropylethylamine was slowly added dropwise to the solution, resulting in the formation of compounds **15a**–**15e**. Then, the glycoside protection was removed by treatment with a methanol/sodium methoxide solution, generating compounds **C1**–**C5**. Further, compounds **C1**–**C5** were dissolved in methanol. A 1N hydrochloric acid solution was slowly added dropwise, and the mixture was stirred at room temperature to obtain compounds **C6**–**C10** (Scheme 3).

### 2.2. Evaluation of the Biological Activity of CE and Its Derivatives

#### 2.2.1. Cytotoxicity Screening of Derivatives

**Figure 2:**
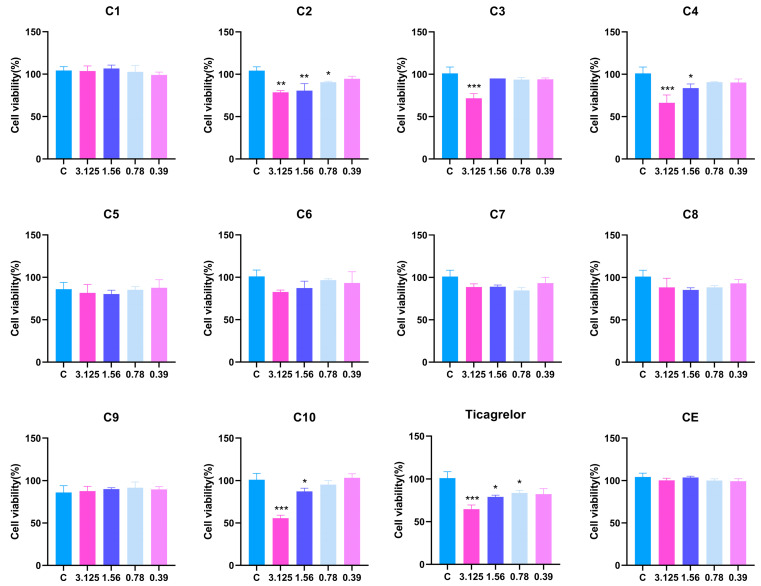
Toxicity assessment of ticagrelor, CE, and CE derivatives (**C1**–**C10**) in HUVECs. Data are presented as mean ± SD of each group (*n* = 3). * *p* < 0.05; ** *p* < 0.01; *** *p* < 0.001 vs. control group.

We evaluated the cytotoxicity and biological activity of the derivatives **C1**–**C10** in human umbilical vein endothelial cells (HUVECs). Initially, a cytotoxicity screening was conducted for these derivatives (Figure 2). Compounds **C2**, **C3**, **C4**, and **C10** exhibited cytotoxicity at a concentration of 3.125 μM. Among them, compound **C2** remained toxic even at a lower concentration of 0.78 μM. Other compounds showed no obvious cytotoxicity at 3.125 μM, making them suitable for further biological activity evaluation.

#### 2.2.2. Protective Effects of CE Derivatives Against Ox-LDL-Induced Damage in HUVECs

**Figure 3:**
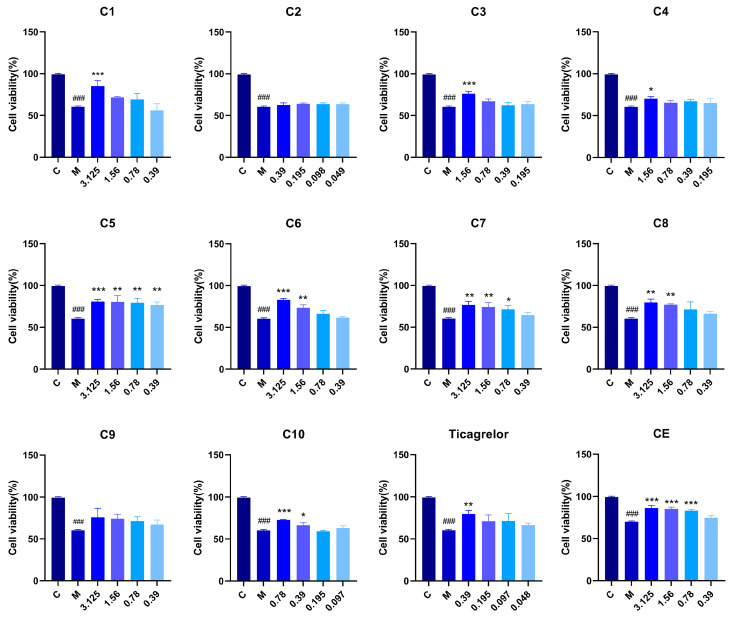
Anti-atherosclerotic activity of **C1**–**C10**, CE, and ticagrelor in ox-LDL-induced injury in HUVECs. Data are presented as mean ± SD of each group (*n* = 3). ^###^ *p* < 0.001 vs. control group; * *p* < 0.05; ** *p* < 0.01; *** *p* < 0.001 vs. model group.

Within the concentration range without cytotoxicity, the activity of Calenduloside E (CE) derivatives was evaluated using the oxidized low-density lipoprotein (ox-LDL)-induced HUVECs injury protection assay (Figure 3). The results showed that when the sugar moiety of the compounds was glucose, derivatives **C1** and **C6** exhibited comparable protective activity on HUVECs. When the sugar moiety was replaced with galactose, xylose, or arabinose, derivatives (**C2**, **C3**, and **C4**) containing the cyclopentyl fused-ring structure of the ticagrelor fragment showed strong cytotoxicity, and their safe concentration range was significantly narrowed. Among the ring-opened derivatives with galactose, xylose, or arabinose as the glycosyl moieties (**C7**, **C8**, **C9**), compound **C9** did not exhibit significant protective activity against ox-LDL-induced HUVECs injury, while the activity levels of compounds **C7** and **C8** were essentially comparable. Among the open-ring derivatives, except that **C9** showed no obvious HUVEC protective activity, **C7** and **C8** had essentially equivalent activity levels. When the sugar moiety was rhamnose, the protective activity of the cyclopentyl fused-ring compound **C5** was significantly superior to that of its corresponding open-ring derivative **C10**. This indicates that the synergistic effect between this sugar moiety and the cyclopentyl fused-ring structure can significantly enhance the biological activity of the compounds. Comprehensive analysis of the cellular activity data of CE derivatives revealed that compound **C5** exhibited the optimal activity, with an effective concentration as low as 0.39 μM, which was superior to the parent compound CE. This low effective concentration provides important experimental basis for reducing dosage in subsequent clinical applications and minimizing potential toxic side effects caused by high-dose exposure. Meanwhile, it also lays a foundation for further structural optimization and druggability evaluation of this type of compounds.

#### 2.2.3. Protective Effects of Compound **C5** Against Ox-LDL-Induced Damage in HUVECs

**Figure 4:**
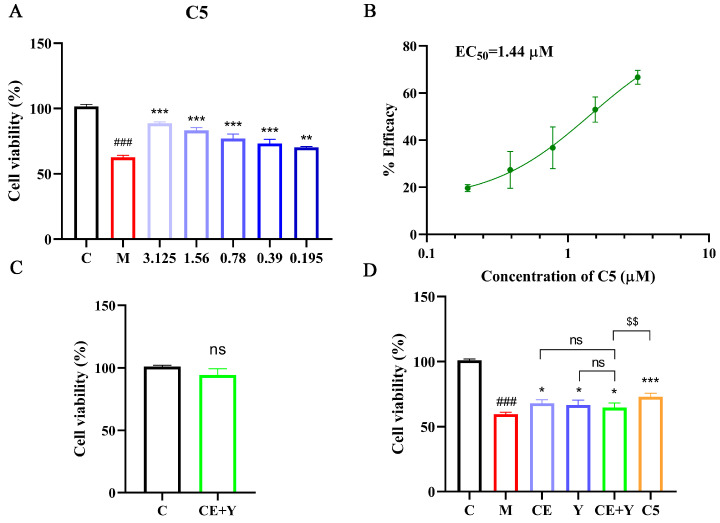
Protective effects of compound **C5** against ox-LDL-induced damage in HUVECs. (**A**) Protective effects of compound **C5** (3.125, 1.56, 0.78, 0.39, and 0.195 μM). Data are presented as mean ± SD of each group (*n* = 3). ^###^ *p* < 0.001 vs. control group, ** *p* < 0.01, *** *p* < 0.001 vs. model group. (**B**) EC_50_ value of **C5** (3.125, 1.56, 0.78, 0.39, and 0.195 μM). (**C**) Toxicity assessment of mixture of CE and ticagrelor (1:1) at 0.39 μM. ns means no significant difference vs. Control group. Data are presented as mean ± SD of each group (*n* = 3). (**D**) Protective effects of compound **C5**, CE, ticagrelor, and mixture of CE and ticagrelor (1:1) at 0.39 μM. Data are presented as mean ± SD of each group (*n* = 3). ^###^ *p* < 0.001 vs. control group; * *p* < 0.05; *** *p* < 0.001 vs. model group; ns means no significant difference vs. CE + Y group; ^$$^ *p* < 0.01 vs. CE + Y group.

Through the comprehensive analysis of the protective activities of CE derivatives, compound **C5** exhibited the optimal efficacy. To determine its half-maximal effective concentration (EC_50_), we expanded the concentration range of compound **C5** for the assay. As shown in Figure 4A, compound **C5** still exerted significant endothelial protective activity within the concentration range of 0.39–3.125 μM. Notably, its protective effect remained statistically significant even when the concentration was reduced to 0.195 μM. A concentration–response curve of compound **C5** against endothelial cell protection rate was plotted using GraphPad Prism 8.0.2 software, and the calculated EC_50_ value of compound **C5** was 1.44 μM (Figure 4B). These findings carry substantial implications for the translational potential of compound **C5** in anti-atherosclerotic drug development.

Compound **C5** was synthesized by conjugating the active fragment of CE with that of ticagrelor. To verify whether covalent linkage between CE and ticagrelor is necessary, we evaluated the endothelial protective activity of a 1:1 mixture of CE and ticagrelor. According to the above cytotoxicity assay results, 0.39 μM (a non-toxic and active concentration for ticagrelor) was used as the concentration in subsequent experiments. First, we conducted a cytotoxicity assessment of this mixture. The results showed that the mixture did not exert significant toxicity on HUVECs at 0.39 μM (Figure 4C). Subsequently, we investigated the protective activity of the mixture against ox-LDL-induced HUVECs injury (Figure 4D and Figure S31). The results demonstrated that the 1:1 mixture of CE and ticagrelor possessed endothelial protective activity. Its protective activity was not significantly different from that of CE or ticagrelor administered alone. However, compared with the conjugated compound **C5**, the CE–ticagrelor mixture exhibited lower protective activity. The enhanced activity of the conjugated compound **C5** suggests that the covalent conjugation strategy contributes to the synergistic improvement in protective efficacy. This may be attributed to covalent linkage optimizing the spatial conformation of the compound, thereby enhancing its affinity with the molecular target (e.g., HSP90β). In addition, the non-significant difference in activity between the mixture and the individual components (CE or ticagrelor) implies that there is no obvious synergistic effect between CE and ticagrelor when they are simply mixed, further highlighting the rationality of the fragment conjugation strategy for constructing compound **C5**. Collectively, these results validate the superiority of the fragment conjugation approach in developing high-activity endothelial protective agents, providing a solid experimental basis for the subsequent optimization and mechanistic research of compound **C5**.

### 2.3. Molecular Docking Analysis of Compound ***C5*** with HSP90β

**Figure 5:**
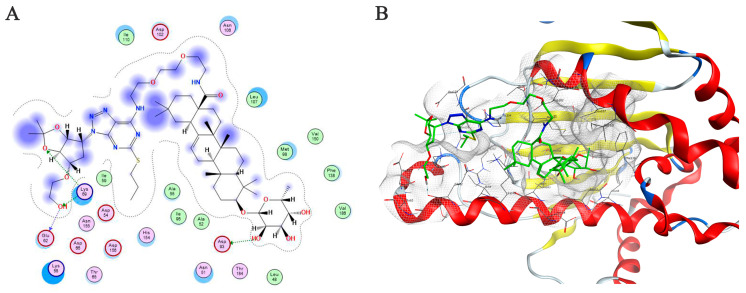
Molecular docking results of HSP90β (PBD ID: 3NMQ) with **C5**. (**A**) Two-dimensional ligand interaction diagram of **C5** and HSP90β. The green arrows represent hydrogen bond interactions. The blue arrow represents electrostatic interaction. (**B**) Three-dimensional modeling of **C5** binding with HSP90β.

In previous studies, we introduced an alkynyl group into the C28 carboxyl group of Calenduloside E (CE) and synthesized a click chemistry-active probe of CE [26]. Through proteomic analysis, heat shock protein 90β (HSP90β) was identified as the core target of CE from HUVECs lysates. To explore whether CE derivative **C5** could still exert protective effects against ox-LDL-induced HUVECs injury by binding to HSP90β, we used Molecular Operating Environment (MOE) to study the interaction between compound **C5** and HSP90β (Figure 5A,B). To explore whether CE derivative **C5** can still exert protective effects against resisting ox-LDL-induced endothelial cell injury by binding to HSP90β, we used Molecular Operating Environment (MOE) to study the interaction between compound **C5** and HSP90β (Figure 5A,B). The 3D structure of HSP90β was retrieved from the Protein Data Bank (PDB ID: 3NMQ). Based on virtual calculation results of the interaction between **C5** and the target HSP90β protein, an S value of −10.61 was obtained. The results showed that **C5** could bind to the N-terminal domain of HSP90β. The hydroxyl group on rhamnose formed a hydrogen bond interaction with the key amino acid residue aspartic acid 93 (Asp93). This result was consistent with previous docking result between CE and HSP90β. In addition, compared with CE, the ticagrelor moiety of derivative **C5** could also form a hydrogen bond with lysine 69 (Lys69) of HSP90β and an electrostatic interaction with glutamic acid 62 (Glu62) of HSP90β. These results provide evidence that compound **C5** exerts the effect of resisting ox-LDL-induced HUVECs injury by targeting HSP90β.

### 2.4. Surface Plasmon Resonance Analysis of CE and ***C5*** Binding to HSP90β

**Figure 6:**
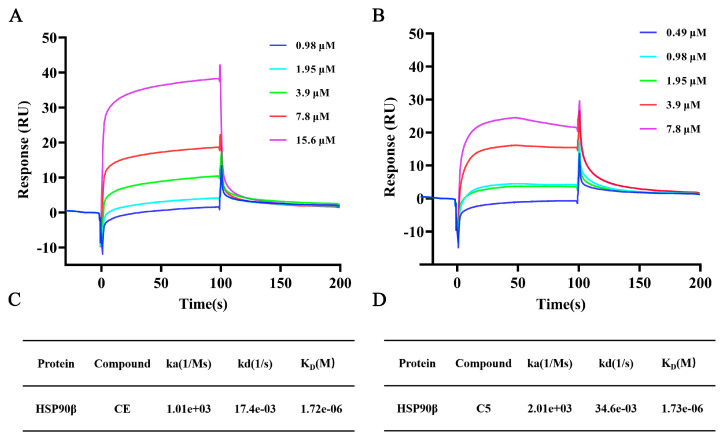
Surface Plasmon Resonance analyses of CE or **C5** binding to HSP90β. (**A**) HSP90β immobilized to a CM5 Sensor Chip was provided with CE at concentrations varying from 0.98 to 15.6 µM. (**B**) HSP90β immobilized to a CM5 Sensor Chip was provided with **C5** at concentrations varying from 0.49 to 7.8 µM. Representative binding curves of CE (**A**) and **C5** (**B**) binding to HSP90β. (**C**,**D**) Kinetic binding constants of CE (**C**)/**C5** (**D**) with HSP90β.

To further verify the target of derivative **C5**, we determined the binding affinity between **C5** and HSP90β using Surface Plasmon Resonance (SPR). SPR results showed that both CE and derivative **C5** could specifically bind to HSP90β. The specific kinetic parameters and comparison results are presented in Figure 6. In terms of the equilibrium dissociation constant (K_D_), the K_D_ value of CE binding to HSP90β was 1.72 × 10^−6^ M, while that of **C5** was 1.73 × 10^−6^ M. The two compounds exhibited comparable binding affinities for HSP90β, with both showing a moderate binding strength. The overall binding affinities of the two compounds to HSP90β were essentially equivalent, both belonging to moderate-intensity binding. From the perspective of the dynamic process of association and dissociation, the association rate constant (ka) of **C5** (2.01 × 10^3^ M^−1^s^−1^) was approximately twice that of CE (1.01 × 10^3^ M^−1^s^−1^). This indicates that the initial rate of complex formation between **C5** and HSP90β is faster. Correspondingly, the dissociation rate constant (kd) of **C5** (34.6 × 10^−3^ s^−1^) was also about twice that of CE (17.4 × 10^−3^ s^−1^). This suggests that the complex formed by **C5** and HSP90β dissociates more rapidly, showing an overall kinetic characteristic of ‘fast association–fast dissociation’. As a molecular chaperone, HSP90β regulates the activity of substrate proteins through dynamic association and dissociation. The moderate binding affinity and dynamic binding characteristics not only avoid the complete inhibition of HSP90β function caused by excessively strong binding but also ensure appropriate regulation of its activity. This result indicates that **C5** may exert protective effects against ox-LDL-induced HUVECs injury by binding to HSP90β through its CE moiety, and that HSP90β may be the key target mediating its functional activity. This result indicates that **C5** may exert protectivity effects against resisting ox-LDL-induced HUVEC injury by binding to HSP90β through its CE moiety, and that HSP90β may be the key target mediating its functional performance.

### 2.5. Microscale Thermophoresis Analysis of ***C5*** and Ticagrelor Binding to HSP90β

**Figure 7:**
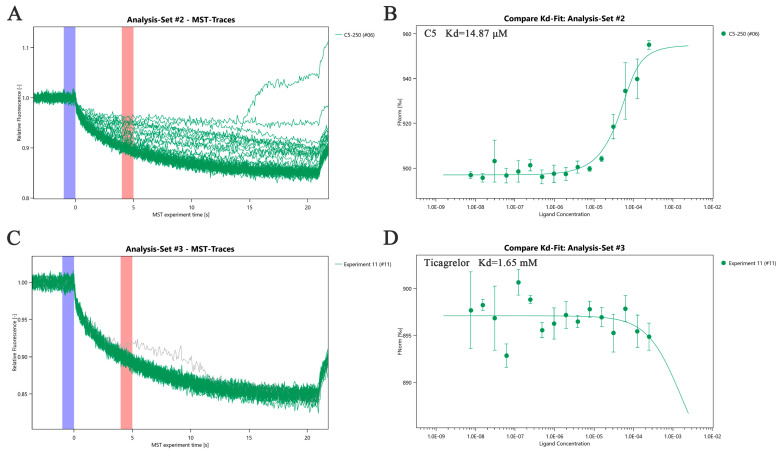
Microscale Thermophoresis analyses of **C5** and ticagrelor binding to HSP90β. (**A**,**B**) Microscale Thermophoresis analyses of **C5** (250 μM) binding to HSP90β (50 nM). (**C**,**D**) Microscale Thermophoresis analyses of ticagrelor (250 μM) binding to HSP90β (50 nM). The blue in (**A**,**C**) represent the initial fluorescence intensity under the laser-off state. The red in (**A**,**C**) represent the fluorescence intensity at thermal steady state when the laser is turned on.

Microscale Thermophoresis (MST) is a biophysical technique used to study molecular interactions [36]. It measures the interaction strength between two molecules by detecting changes in fluorescence signals caused by temperature variations induced by an infrared laser. SPR analysis revealed that the binding affinity of derivative **C5** toward HSP90β was comparable to that of its parent compound CE. SPR results showed that the binding ability of derivative **C5** to HSP90β was essentially equivalent to that of the parent compound CE. Considering that the ticagrelor fragment was introduced into the structural design of **C5**, we further determined the binding affinities of derivative **C5** and ticagrelor monomer to HSP90β via MST. This was aimed at clarifying whether this structural modification exerts a positive regulatory effect on the binding ability between **C5** and HSP90β and at further elucidating the molecular binding mechanism. In the experiment, His-tagged recombinant HSP90β was used as the target molecule, while **C5** and ticagrelor were used as ligands, respectively. A series of concentration gradients were constructed by twofold serial dilution starting from 250 μM, and the interaction detection was performed in an optimized buffer system. MST results showed that the dissociation constant (Kd) of derivative **C5** binding to HSP90β was 14.87 μM, indicating a moderate-intensity specific binding between them (Figure 7A,B). In contrast, the ticagrelor monomer exhibited extremely weak binding ability to HSP90β, with a Kd value of only 1.65 mM (Figure 7C,D). This binding affinity was approximately 111 times lower than that of **C5** to HSP90β. Combining the SPR results and MST data, we speculate that the ticagrelor fragment may not enhance the biological activity by strengthening binding to HSP90β. Combining the SPR results and MST data, we speculate that the ticagrelor fragment introduced into derivative **C5** may not enhance the biological activity by directly strengthening binding to HSP90β. The protective effect of **C5** against ox-LDL-induced HUVECs injury is more likely to rely on other molecular targets or regulatory pathways.

### 2.6. Identification of P2Y12 as Another Potential Target of Derivative ***C5***

**Figure 8:**
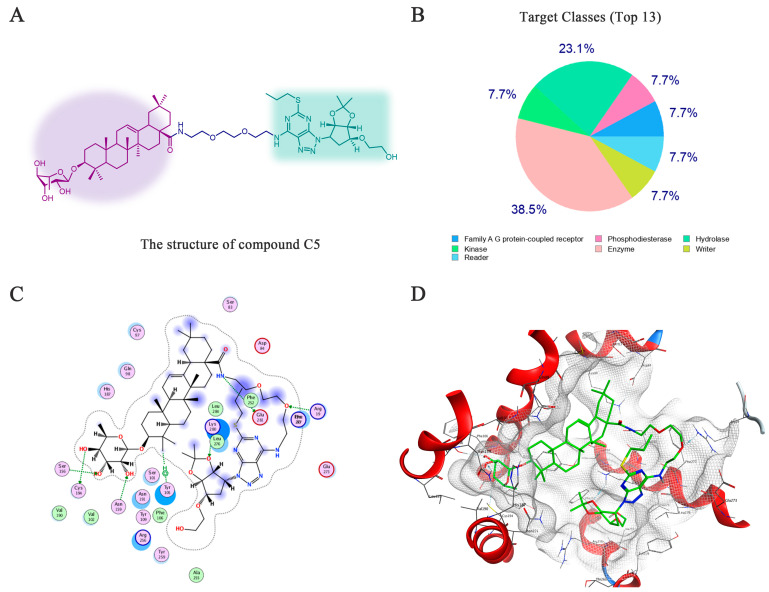
Two-dimensional and three-dimensional depictions of the predicted binding modes of **C5** in the active site of P2Y12 (PDB ID: 4NTJ). (**A**) Structure of compound **C5**. (**B**) Top 13 predicted target classes. The values represent the number of targets. (**C**) Two-dimensional ligand interaction diagram of **C5** and P2Y12. The green arrows represent hydrogen bond interactions. (**D**) Three-dimensional modeling of **C5** binding with P2Y12.

Although ticagrelor is a well-established P2Y12 inhibitor and derivative **C5** retains a ticagrelor-derived moiety, the ticagrelor-derived active fragment in **C5** differs structurally from free ticagrelor. Compared with free ticagrelor, the two hydroxyl groups in the cyclopentane moiety of **C5** are not exposed. This structural difference may affect the ability of **C5** to target P2Y12. Therefore, we performed further predictions using SwissTargetPrediction to confirm the potential of **C5** to target P2Y12. We performed target prediction using the SwissTargetPrediction online database (http://swisstargetprediction.ch/, accessed on 17 November 2025). The top 13 possible biological targets were listed (Figure 8A,B, Table 1), among which P2Y12 was identified as the most likely potential target.

The P2Y12 receptor was initially discovered in platelets. It couples with Gi proteins to inhibit adenylate cyclase activity and reduce cyclic adenosine monophosphate (cAMP) levels. This further activates the PI3K-AKT pathway, thereby regulating platelet aggregation and thrombus stability [37]. With in-depth research, the expression of P2Y12 in vascular walls and immune cells has been gradually confirmed. In VSMCs, P2Y12 can be transcriptionally activated by atherogenic factors such as ox-LDL and thromboxane via the NF-κB pathway [38,39]. It is highly expressed in VSMCs of human carotid plaques, especially enriched near the necrotic core of plaques [40]. In vascular endothelial cells, P2Y12 is involved in regulating endothelial permeability and secretion of inflammatory factors [41]. In immune cells such as monocytes and macrophages, P2Y12 mediates cell chemotaxis and inflammatory responses [42]. Collectively, these cell types constitute the key targets through which P2Y12 affects atherosclerosis.

Based on the above data, we used MOE to study the interaction between compound **C5** and P2Y12. The 3D structure of P2Y12 was retrieved from the Protein Data Bank (PDB ID: 4NTJ) (Figure 8C,D). According to virtual calculation results of the interaction between **C5** and the target P2Y12 protein, an S value of −12.79 was obtained. The results showed that the hydroxyl groups on the rhamnose moiety of derivative **C5** could form hydrogen bond interactions with Asn159, Ser156, and Cys194. The amide linker of the connecting chain formed a hydrogen bond with Glu281, while the oxygen atom of the connecting chain could form a hydrogen bond with Arg19. The cyclopentane oxygen atom in the ticagrelor moiety could form a hydrogen bond with Lys280. In addition, derivative **C5** could also form a π-π stacking interaction with Tyr105. In summary, we speculate that P2Y12 is most likely another potential target through which **C5** exerts its protective effect against ox-LDL-induced HUVEC injury, providing a basis for the further rational design of derivatives in the future. The ticagrelor-derived moiety in derivative **C5** is predicted to bind the P2Y12 receptor, which is the well-established target of ticagrelor. This is a logical and expected outcome of our molecular hybridization design. In developing CE–ticagrelor hybrid derivatives, our core strategy was to integrate the active cyclopentyl fused-ring fragment of ticagrelor with CE’s anti-atherosclerotic scaffold. The design was based on a key hypothesis: retaining ticagrelor’s P2Y12-binding motif would allow the hybrid derivatives to engage P2Y12 while harnessing CE’s inherent biological activity, ultimately achieve synergistic anti-atherosclerotic effects. The target prediction and molecular docking results for P2Y12 not only validate the rationality of our hybridization strategy, but also provide a structural basis for understanding the dual-targeting potential of derivative **C5**.

## 3. Materials and Methods

### 3.1. Chemistry

#### 3.1.1. General Experiment and Information

All commercially available reagents were used without further purification. Reaction were monitored by GF254 thin-layer chromatography (TLC) on prefabricated silica gel plate from Qingdao Haiyang Chemical Co., Ltd. (Qingdao, China). Synthetic compounds were purified using silica gel column chromatography (200–300 mesh). After reaction and extraction, solutions were concentrated and dried using a rotary evaporator. ^1^H-NMR and ^13^C-NMR spectra were recorded on a Bruker AvanceIII 600 MHz spectrometer (Bruker Corporation, Karlsruhe, Germany). HRMS was performed on a Thermo Fisher LTQ Orbitrap XL (Thermo Fisher Scientific, Waltham, MA, USA). ^1^H-NMR, ^13^C-NMR spectra, and HRMS data are shown in Figures S1–S30.

#### 3.1.2. General Procedure for the Synthesis of **5a**–**5e**

Glycosyl donor **5a**–**5e** was prepared from glucose (**1a**), galactose (**1b**), xylose (**1c**), arabinose (**1d**), and rhamnose (**1e**) under reaction conditions reported previously by Schmidt and Michel [32] (Scheme 1).

#### 3.1.3. Synthesis of Compound **7**

To a solution of oleanolic acid (**6**) (20.0 g, 43.8 mmol) in dichloromethane (DCM) (400 mL), tetrabutylammonium bromide (TBAB) (1.6 g, 4.9 mmol) and K_2_CO_3_ (14.8 g, 107.2 mmol) in water (50 mL) were added, and benzyl bromide (6.4 mL, 54.3 mmol) was dropped at 0 °C. Then, the reaction mixture was stirred at room temperature for 18 h and monitored by TLC. After the reaction was completed, the mixture was extracted with DCM (3 × 100 mL) and water. The crude mixture was separated, and the aqueous layer was extracted with DCM (3 × 100 mL). The combined organic layer was washed with 0.1 mol/L HCl aqueous solution, NaHCO_3_ saturated aqueous solution, and NaCl saturated aqueous solution in sequence, then dried over Na_2_SO_4_ and purified through column chromatography (PE:EA = 8:1) to afford compound **7** as a pure white solid (18.3 g, 76.4% yield).

#### 3.1.4. General Procedure for the Synthesis of **8a**–**8e**

To a solution of compound **7** (3.3 g, 6.0 mmol) and glycosyl donors (**5a**: 5.8 g, 7.9 mmol; **5b**: 5.0 g, 7.9 mmol; **5c**: 4.8 g, 7.9 mmol; **5d**: 4.8 g, 7.9 mmol; **5e**: 5.0 g, 7.9 mmol) in anhydrous DCM (50 mL) was added 4 Å molecular sieves (5.0 g). The mixture was stirred at room temperature for 1 h under N_2_.To a solution of compound **7** (3.3 g, 6.0 mmol) and glycosyl donor (**5a**: 5.8 g, 7.9 mmol; **5b**: 5.0 g, 7.9 mmol; **5c**: 4.8 g, 7.9 mmol; **5d**, 4.8 g, 7.9 mmol; **5e**: 5.0 g, 7.9 mmol) in dry DCM (50 mL), 4 A molecular sieve (5.0 g) were added, and the mixture was stirred at room temperature for 1 h under N_2_. Subsequently, the TMSOTf (60 μL, 0.3 mmol) was added dropwise, and the reaction mixture was stirred for a further 2–4 h. Then Lewis acid TMSOTf (60 μL, 0.3 mmol) was dropwise, and the mixture reacted for 2–4 h. Upon completion, triethylamine 1.0 mL was added to stop the reaction. Then, the suspension was filtered, the filtrate was evaporated, and the crude product was subjected to column chromatography (petroleum ether, PE/ethyl acetate, EA = 10:1) to gain pure compounds **8a** (7.0 g, 79.5% yield), **8b** (7.3 g, 83.0% yield), **8c** (6.4 g, 82.1% yield), **8d** (6.9 g, 88.5% yield), and **8e** (6.7 g, 83.8% yield) as white solids.

#### 3.1.5. General Procedure for the Synthesis of **9a**–**9e**

A solution of **8a**–**8e** (3.0 mmol) and 10% Pd/C (2.0 g) in EA (100 mL) was hydrogenated at 1 atm for 4–6 h under reflux. The mixture was filtered and concentrated, and the residue was purified by silica gel column chromatography (PE:EA = 3:1) to obtain pure compounds **9a** (2.6 g, 83.3% yield), **9b** (2.8 g, 89.7% yield), **9c** (2.4 g, 88.2% yield), **9d** (2.5 g, 91.9% yield), and **9e** (2.3 g, 84.6% yield).

#### 3.1.6. General Procedure for the Synthesis of **10a**–**10e**

To a solution of **9a**–**9e** (1.0 mmol) in dry DCM (15 mL), 1-hydroxybenzotriazole (HOBT) (0.13 g, 1.0 mmol) and 1-ethyl-3- (3-dimethyllaminopropyl) carbodiimide hydrochloride (EDCI) (0.19 g, 1.0 mmol) were added, and the mixture was stirred at room temperature for 1 h. Then 1,8-diamino-3,6-dioxaoctane (10.0 mmol) was added at 0 °C, and the reaction mixture was stirred for 4 h until completion. To this mixture, 1,8-diamino-3,6-dioxaoctane (10.0 mmol) was added at 0 °C, and the reaction mixture was stirred for 4 h until completion. The solvent was washed with 0.1 mol/L HCl aqueous solution, NaHCO_3_ saturated aqueous solution, and NaCl saturated aqueous solution in sequence, then dried over Na_2_SO_4_. The suspension was filtered and the filtrate was concentrated. The residue was purified by silica gel column chromatography (PE:EA = 8:1) to obtain pure compounds **10a** (0.80 g, 68.9% yield), **10b** (0.89 g, 76.7% yield), **10c** (0.73 g, 70.9% yield), **10d** (0.69 g, 67.0% yield), and **10e** (0.75 g, 71.8% yield).

#### 3.1.7. Synthesis of Compound **13**

A solution of ticagrelor tartrate (5.0 g, 21.20 mmol) and 4,6-dichloro-2-propylthiopyrimidine-5-amine (9.3 g, 25.23 mmol) in 1,4-dioxane (150 mL) was stirred at room temperature for 5 min. Then, triethylamine (12.2 g, 10.59 mmol) was added, and the mixture was heated under reflux at 110 °C with stirring for 24 h. The solvent was washed with 0.1 mol/L HCl aqueous solution, NaHCO_3_ saturated aqueous solution, and NaCl saturated aqueous solution in sequence, then dried over Na_2_SO_4_. The suspension was filtered and the filtrate was concentrated. The residue was purified by silica gel column chromatography (PE:EA = 4:1) to obtain pure compound **13** (7.3 g, 56.9% yield).

#### 3.1.8. Synthesis of Compound **14**

A solution of compound **13** (7.0 g, 16.7 mmol) and acetic acid (5.6 mL, 100.2 mmol) in ethyl acetate (100 mL) was stirred at room temperature. Subsequently, the aqueous NaNO_2_ solution (20 mL) was added dropwise, and the reaction mixture was maintained in an ice bath for a further 8 h. Then, 20 mL of NaNO_2_ aqueous solution was added dropwise with stirring, and the mixture was kept in an ice bath for 8 h. The reaction solution was adjusted to pH 8 with saturated K_2_CO_3_ aqueous, washed with NaCl saturated aqueous solution, and then dried over Na_2_SO_4_. The suspension was filtered and the filtrate was concentrated. The residue was purified by silica gel column chromatography (PE:EA = 4:1) to obtain pure compound **14** (5.9 g, 81.9% yield).

#### 3.1.9. General Procedure for the Synthesis of **15a**–**15e**

To a solution of **10a**–**10e** (0.60 mmol) and compound **14** (0.26 g, 0.60 mmol) in DCM (100 mL), N,N-diisopropylethylamine (0.66 mmol) was added. The mixture was stirred at 10 °C for 10 h. The solvent was washed with 1 mol/L HCl aqueous solution and NaCl saturated aqueous solution in sequence, and then dried over Na_2_SO_4_. The suspension was filtered and the filtrate was concentrated. The residue was purified by silica gel column chromatography (DCM:CH_3_OH = 20:1) to obtain pure compounds **15a** (0.61 g, 66.3% yield), **15b** (0.54 g, 58.7% yield), **15c** (0.51 g, 59.3% yield), **15d** (0.47 g, 54.6% yield), and **15e** (0.49 g, 56.3% yield).

#### 3.1.10. General Procedure for the Synthesis of **C1**–**C5**

To a solution of compounds **15a**–**15e** (0.30 mmol) in CH_3_OH/DCM (8.0 mL, 3:1), 1 mol/L sodium methoxide/CH_3_OH solvent (2 mL) was added. The reaction mixture was stirred for 2–3 h until the reaction was completed, after which Amberlite IR-120 cation-exchange resin was added to adjust the pH to 7. The reaction mixture was stirred for 2–3 h until completion, after which Amberlite IR-120 was added to adjust the pH to pH = 7. The suspension was filtered, and the filtrate was evaporated and purified through column chromatography (DCM:CH_3_OH = 10:1) to afford pure white solids **C1** (267 mg, 78.1% yield), **C2** (281 mg, 82.2% yield), **C3** (274 mg, 82.0% yield), **C4** (271 mg, 81.1% yield), and **C5** (263 mg, 78.0% yield).

#### 3.1.11. Characterization of **C1**–**C5**

(4aS,6aS,6bR,10S,12aR)-N-(2-(2-(2-((3-((3aS,6S,6aR)-6-(2-hydroxyethoxy)-2,2-dimethyltetrahydro-4H-cyclopenta[d][1,3]dioxol-4-yl)-5-(propylthio)-3H-[1,2,3]triazolo[4,5-d]pyrimidin-7-yl)amino)ethoxy)ethoxy)ethyl)-2,2,6a,6b,9,9,12a-heptamethyl-10-(((2R,3R,5S,6R)-3,4,5-trihydroxy-6-(hydroxymethyl)tetrahydro-2H-pyran-2-yl)oxy)-1,3,4,5,6,6a,6b,7,8,8a,9,10,11,12,12a,12b,13,14b-octadecahydropicene-4a(2H)-carboxamide (Compound **C1**), 267 mg, yield is 78.1%, white solid. ^1^H-NMR (600 MHz, pyridine-*d*5) *δ*: 10.23–10.19 (m, 1H), 7.48–7.46 (m, 1H), 5.62–5.60 (m, 1H), 5.46–5.41 (m, 2H), 4.99–4.97 (m, 2H), 4.64–4.62 (m, 1H), 4.50–4.45 (m, 1H), 4.32–4.24 (m, 3H), 4.12–4.04 (m, 4H), 4.02–3.97 (m, 2H), 3.92 (dt, J = 13.5, 5.3 Hz, 3H), 3.81 (dt, J = 9.9, 5.0 Hz, 1H), 3.78–3.71 (m, 3H), 3.69–3.59 (m, 6H), 3.42 (dd, J = 11.8, 4.3 Hz, 1H), 3.27–3.15 (m, 2H), 3.08–3.05 (m, 1H), 2.98–2.91 (m, 1H), 2.87–2.83 (m, 1H), 1.62 (s, 3H), 1.36 (s, 6H), 1.29 (s, 3H), 1.08 (t, J = 7.4 Hz, 3H), 1.04 (s, 3H), 0.96 (s, 3H), 0.94 (s, 3H), 0.93 (s, 3H), 0.91 (s, 3H). ^13^C-NMR (150 MHz, pyridine-*d*5) *δ*: 178.18, 171.12, 154.88, 145.27, 124.97, 113.65, 107.44, 89.26, 85.30, 83.87, 83.42, 79.25, 78.82, 76.28, 72.64, 72.28, 71.24, 71.06, 70.68, 70.41, 63.50, 62.81, 62.01, 56.19, 48.37, 47.22, 46.93, 42.61, 42.37, 41.56, 40.31, 40.22, 39.99, 39.18, 37.41, 37.02, 34.84, 34.13, 33.90, 33.67, 33.44, 31.35, 28.71, 28.27, 27.69, 27.05, 26.56, 25.42, 24.30, 24.21, 24.16, 24.00, 18.92, 17.85, 17.51, 16.02, 14.25. HRMS (ESI) calculated for C_59_H_95_N_7_NaO_13_S [M + Na]^+^: 1164.6606, found 1164.6606.

(4aS,6aS,6bR,10S,12aR)-N-(2-(2-(2-((3-((3aS,6S,6aR)-6-(2-hydroxyethoxy)-2,2-dimethyltetrahydro-4H-cyclopenta[d][1,3]dioxol-4-yl)-5-(propylthio)-3H-[1,2,3]triazolo[4,5-d]pyrimidin-7-yl)amino)ethoxy)ethoxy)ethyl)-2,2,6a,6b,9,9,12a-heptamethyl-10-(((2R,3R,5R,6R)-3,4,5-trihydroxy-6-(hydroxymethyl)tetrahydro-2H-pyran-2-yl)oxy)-1,3,4,5,6,6a,6b,7,8,8a,9,10,11,12,12a,12b,13,14b-octadecahydropicene-4a(2H)-carboxamide (Compound **C2**), 281 mg, yield is 82.2%, white solid. ^1^H-NMR (600 MHz, pyridine-*d*5) *δ*: 10.21 (t, J = 6.0 Hz, 1H), 7.48–7.46 (m, 1H), 5.61–5.59 (m, 1H), 5.46–5.45 (m, 1H), 5.44–5.41 (m, 1H), 4.98–4.97 (m, 1H), 4.90 (d, J = 6.0 Hz, 1H), 4.63 (s, 1H), 4.57–4.45 (m, 3H), 4.29–4.25 (m, 1H), 4.23–4.20 (m, 1H), 4.18–4.15 (m, 1H), 4.09–4.05 (m, 2H), 3.99 (s, 2H), 3.95–3.92 (m, 1H), 3.92–3.91 (m, 2H), 3.82–3.80 (m, 1H), 3.78–3.72 (m, 3H), 3.70–3.57 (m, 6H), 3.42 (dd, J = 12.0, 6.0 Hz, 1H), 3.25–3.16 (m, 2H), 3.06 (m, J = 13.3, 4.6 Hz, 1H), 2.97–2.91 (m, 1H), 2.87–2.82 (m, 1H), 1.62 (s, 3H), 1.36 (s, 3H), 1.35 (s, 3H), 1.29 (s, 3H), 1.08 (t, J = 7.3 Hz, 3H), 1.01 (s, 3H), 0.98 (s, 1H), 0.96 (s, 3H), 0.94 (s, 3H), 0.92 (s, 3H). ^13^C-NMR (150 MHz, pyridine-*d*5) *δ*: 178.19, 171.12, 154.88, 145.26, 124.97, 113.66, 108.07, 89.14, 85.30, 83.86, 83.42, 77.32, 75.96, 73.66, 72.65, 71.24, 71.07, 70.75, 70.68, 70.41, 62.91, 62.81, 62.01, 56.22, 48.39, 47.22, 46.93, 42.61, 42.36, 41.57, 40.31, 40.23, 40.01, 39.23, 37.42, 37.02, 34.85, 34.13, 33.91, 33.67, 33.46, 31.36, 28.70, 28.27, 27.69, 27.15, 26.57, 25.42, 24.31, 24.21, 24.16, 24.00, 18.92, 17.85, 17.48, 16.03, 14.25. HRMS (ESI) calculated for C_59_H_95_N_7_NaO_13_S [M + Na]^+^: 1164.6606, found 1164.6606.

(4aS,6aS,6bR,10S,12aR)-N-(2-(2-(2-((3-((3aS,6S,6aR)-6-(2-hydroxyethoxy)-2,2-dimethyltetrahydro-4H-cyclopenta[d][1,3]dioxol-4-yl)-5-(propylthio)-3H-[1,2,3]triazolo[4,5-d]pyrimidin-7-yl)amino)ethoxy)ethoxy)ethyl)-2,2,6a,6b,9,9,12a-heptamethyl-10-(((2S,3R,5R)-3,4,5-trihydroxytetrahydro-2H-pyran-2-yl)oxy)-1,3,4,5,6,6a,6b,7,8,8a,9,10,11,12,12a,12b,13,14b-octadecahydropicene-4a(2H)-carboxamide (Compound **C3**), 274 mg, yield is 82.0%, white solid. ^1^H-NMR (600 MHz, pyridine-*d*5) *δ*: 10.21 (t, J = 5.9 Hz, 1H), 7.47–7.43 (m, H), 5.62–5.58 (m, 1H), 5.47–5.40 (m, 2H), 4.98–4.95 (m, 1H), 4.87 (d, J = 7.6 Hz, 1H), 4.45–4.40 (m, 1H), 4.30–4.25 (m, 2H), 4.21 (t, J = 8.8 Hz, 1H), 4.09–4.04 (m, 3H), 3.99 (s, 2H), 3.96–3.90 (m, 3H), 3.85–3.79 (m, 2H), 3.78–3.73 (m, 3H), 3.69–3.58 (m, 6H), 3.38 (dd, J = 11.8, 4.4 Hz, 1H), 3.25–3.16 (m, 2H), 3.05 (dd, J = 13.6, 4.6 Hz, 1H), 2.97–2.91 (m, 1H), 2.88–2.82 (m, 1H), 1.62 (s, 3H), 1.36 (s, 3H), 1.35 (s, 3H), 1.28 (s, 3H), 1.08 (t, J = 7.4 Hz, 3H), 1.03 (s, 3H), 0.97 (s, 3H), 0.95 (s, 3H), 0.93 (s, 6H). ^13^C-NMR (150 MHz, pyridine-*d*5) δ 178.17, 171.12, 154.88, 145.20, 124.97, 113.65, 108.23, 89.06, 85.29, 83.86, 83.42, 79.15, 76.05, 72.65, 71.72, 71.24, 71.06, 70.68, 70.42, 67.64, 62.80, 62.01, 56.23, 48.42, 47.20, 46.93, 42.60, 42.37, 41.56, 40.31, 40.24, 40.06, 39.25, 37.45, 37.01, 34.83, 34.12, 33.90, 33.67, 33.45, 31.34, 28.64, 28.26, 27.69, 27.22, 26.51, 25.42, 24.32, 24.22, 24.16, 24.00, 18.92, 17.85, 17.44, 16.05, 14.25. HRMS (ESI) calculated for C_58_H_93_N_7_NaO_12_S [M + Na]^+^: 1134.6501, found 1134.6505.

(4aS,6aS,6bR,10S,12aR)-N-(2-(2-(2-((3-((3aS,6S,6aR)-6-(2-hydroxyethoxy)-2,2-dimethyltetrahydro-4H-cyclopenta[d][1,3]dioxol-4-yl)-5-(propylthio)-3H-[1,2,3]triazolo[4,5-d]pyrimidin-7-yl)amino)ethoxy)ethoxy)ethyl)-2,2,6a,6b,9,9,12a-heptamethyl-10-(((2S,3S,4R,5R)-3,4,5-trihydroxytetrahydro-2H-pyran-2-yl)oxy)-1,3,4,5,6,6a,6b,7,8,8a,9,10,11,12,12a,12b,13,14b-octadecahydropicene-4a(2H)-carboxamide (Compound **C4**), 271 mg, yield is 81.1%, white solid. ^1^H-NMR (600 MHz, pyridine-*d*5) *δ*: 10.22 (t, J = 5.6 Hz, 1H), 7.48–7.44 (m, 1H), 5.63–5.58 (m, 1H), 5.47–5.40 (m, 2H), 4.99–4.93 (m, 2H), 4.80 (d, J = 7.1 Hz, 1H), 4.49–4.45 (m, 1H), 4.38–4.34 (m, 2H), 4.29–4.25 (m, 1H), 4.23–4.18 (m, 1H), 4.11–4.04 (m, 2H), 3.99 (s, 2H), 3.95–3.90 (m, 3H), 3.89–3.85 (m, 1H), 3.83–3.79 (m, 1H), 3.78–3.72 (m, 3H), 3.70–3.58 (m, 6H), 3.38 (dd, J = 11.8, 4.4 Hz, 1H), 3.24 (td, J = 7.1, 2.2 Hz, 2H), 3.06 (dd, J = 12.8, 4.4 Hz, 1H), 2.98–2.91 (m, 1H), 2.85 (dt, J = 13.1, 6.6 Hz, 1H), 1.62 (s, 3H), 1.37 (s, 3H), 1.32 (s, 3H), 1.28 (s, 3H), 1.08 (t, J = 7.4 Hz, 3H), 1.00 (s, 3H), 0.97 (s, 3H), 0.95 (s, 3H), 0.94 (s, 6H). ^13^C-NMR (150 MHz, pyridine-*d*5) *δ* 177.48, 170.43, 154.20, 144.51, 124.28, 122.64, 112.96, 107.43, 88.40, 84.61, 83.17, 82.73, 74.46, 72.73, 71.96, 70.56, 70.38, 69.99, 69.73, 69.40, 66.67, 62.12, 61.32, 55.56, 47.74, 46.51, 46.24, 41.91, 41.68, 40.88, 39.62, 39.54, 39.36, 38.57, 36.76, 36.33, 34.15, 33.44, 33.21, 32.98, 32.77, 30.66, 29.77, 27.99, 27.58, 27.00, 26.46, 25.83, 24.73, 23.64, 23.52, 23.47, 23.31, 18.24, 17.17, 16.72, 15.36, 13.57. HRMS (ESI) calculated for C_58_H_93_N_7_NaO_12_S [M + Na]^+^: 1134.6501, found 1134.6505.

(4aS,6aS,6bR,10S,12aR)-N-(2-(2-(2-((3-((3aS,6S,6aR)-6-(2-hydroxyethoxy)-2,2-dimethyltetrahydro-4H-cyclopenta[d][1,3]dioxol-4-yl)-5-(propylthio)-3H-[1,2,3]triazolo[4,5-d]pyrimidin-7-yl)amino)ethoxy)ethoxy)ethyl)-2,2,6a,6b,9,9,12a-heptamethyl-10-(((2R,3R,4R,5R,6S)-3,4,5-trihydroxy-6-methyltetrahydro-2H-pyran-2-yl)oxy)-1,3,4,5,6,6a,6b,7,8,8a,9,10,11,12,12a,12b,13,14b-octadecahydropicene-4a(2H)-carboxamide (Compound **C5**), 263 mg, yield is 78.0%, white solid. ^1^H-NMR (600 MHz, pyridine-*d*5) *δ*: 10.23–10.19 (m, 1H), 7.48–7.43 (m, 1H), 5.62–5.59 (m, 1H), 5.48–5.41 (m, 2H), 4.62 (s, 1H), 4.53–4.49 (m, 1H), 4.39–4.32 (m, 2H), 4.28–4.25 (m, 1H), 4.09–4.05 (m, 2H), 4.01–3.97 (m, 2H), 3.94–3.88 (m, 3H), 3.83–3.79 (m, 1H), 3.79–3.74 (m, 3H), 3.69–3.59 (m, 6H), 3.27–3.22 (m, 2H), 3.19 (dd, J = 11.7, 4.5 Hz, 1H), 3.09–3.02 (m, 1H), 2.97–2.91 (m, 1H), 2.88–2.82 (m, 1H), 1.71 (d, J = 5.8 Hz, 3H), 1.62 (s, 3H), 1.37 (s, 3H), 1.26 (s, 3H), 1.09 (t, J = 7.4 Hz, 3H), 0.97 (s, 3H), 0.96 (s, 3H), 0.94 (s, 3H), 0.94 (s, 3H), 0.93 (s, 3H), 0.84 (s, 3H). ^13^C-NMR (150 MHz, pyridine-*d*5) *δ* 178.17, 171.12, 154.89, 145.24, 124.97, 113.65, 104.96, 88.95, 85.29, 83.86, 83.42, 74.60, 73.41, 72.98, 72.65, 71.25, 71.08, 70.69, 70.43, 70.35, 62.80, 62.01, 55.93, 48.36, 47.20, 46.93, 42.58, 42.37, 41.57, 40.31, 40.20, 39.64, 39.00, 37.39, 37.04, 34.84, 34.13, 33.90, 33.66, 33.37, 31.35, 28.72, 28.26, 27.69, 26.52, 26.31, 25.42, 24.30, 24.21, 24.15, 24.00, 19.01, 17.83, 17.21, 15.98, 14.25. HRMS (ESI) calculated for C_59_H_95_N_7_NaO_12_S [M + Na]^+^: 1148.6657, found 1148.6655.

#### 3.1.12. General Procedure for the Synthesis of **C6**–**C10**

To a solution of compounds **C1**–**C5** (0.2 mmol) in CH_3_OH (20.0 mL), 1 mol/L HCl solvent (2 mL) was added, and the mixture were stirred at room temperature for 2 h. After the reaction was completed, the mixture was extracted with EA (3 × 100 mL) and water. The crude mixture was separated, and the aqueous layer was extracted with EA (3 × 100 mL). The reaction solution was adjusted to pH 8 with saturated K_2_CO_3_ aqueous, and was washed with NaCl saturated aqueous solution, and then dried over Na_2_SO_4_. The suspension was filtered, and the filtrate was evaporated and purified through column chromatography (DCM:CH_3_OH = 15:1) to afford pure white solids **C6** (189 mg, 85.9% yield), **C7** (183 mg, 83.2% yield), **C8** (175 mg, 81.8% yield), **C9** (189 mg, 88.3% yield), and **C10** (164 mg, 75.6% yield).

#### 3.1.13. Characterization of **C6**–**C10**

(4aS,6aS,6bR,10S,12aR)-N-(2-(2-(2-((3-((2S,3S,4S)-2,3-dihydroxy-4-(2-hydroxyethoxy)cyclopentyl)-5-(propylthio)-3H-[1,2,3]triazolo[4,5-d]pyrimidin-7-yl)amino)ethoxy)ethoxy)ethyl)-2,2,6a,6b,9,9,12a-heptamethyl-10-(((2R,3R,5S,6R)-3,4,5-trihydroxy-6-(hydroxymethyl)tetrahydro-2H-pyran-2-yl)oxy)-1,3,4,5,6,6a,6b,7,8,8a,9,10,11,12,12a,12b,13,14b-octadecahydropicene-4a(2H)-carboxamide (Compound **C6**), 189 mg, yield is 85.9%, white solid. ^1^H-NMR (600 MHz, pyridine-*d*5) *δ*: 10.16–10.13 (m, 1H), 7.48 (t, J = 5.5 Hz, 1H), 5.83–5.77 (m, 1H), 5.50–5.44 (m, 2H), 4.99–4.97 (m, 1H), 4.80 (s, 1H), 4.63 (d, J = 11.6 Hz, 1H), 4.49–4.44 (m, 1H), 4.42–4.38 (m, 1H), 4.32–4.25 (m, 2H), 4.10–4.04 (m, 6H), 3.99–3.95 (m, 1H), 3.93–3.88 (m, 3H), 3.77–3.72 (m, 3H), 3.71–3.58 (m, 6H), 3.42 (dd, J = 11.8, 4.4 Hz, 1H), 3.28–3.16 (m, 2H), 3.06 (dd, J = 13.3, 4.6 Hz, 1H), 3.03–2.96 (m, 1H), 2.75–2.69 (m, 1H), 1.35 (s, 3H), 1.29 (s, 3H), 1.07–1.03 (m, 6H), 0.96 (s, 4H), 0.94 (s, 3H), 0.93 (s, 3H), 0.91 (s, 3H). ^13^C-NMR (150 MHz, pyridine-*d*5) *δ* 178.21, 170.82, 154.84, 145.27, 124.99, 107.44, 89.27, 83.96, 79.25, 78.83, 76.70, 76.29, 75.94, 72.89, 72.28, 71.25, 71.06, 70.68, 70.45, 63.49, 62.62, 62.28, 56.19, 48.38, 47.22, 46.94, 42.61, 42.37, 41.52, 40.31, 40.23, 39.99, 39.18, 37.41, 34.99, 34.84, 34.13, 33.85, 33.67, 33.45, 31.35, 28.71, 28.28, 27.05, 26.57, 24.30, 24.21, 24.16, 23.91, 18.93, 17.86, 17.52, 16.03, 14.16. HRMS (ESI) calculated for C_56_H_91_N_7_NaO_13_S [M + Na]^+^: 1124.6293, found 1124.6300.

(4aS,6aS,6bR,10S,12aR)-N-(2-(2-(2-((3-((2S,3S,4S)-2,3-dihydroxy-4-(2-hydroxyethoxy)cyclopentyl)-5-(propylthio)-3H-[1,2,3]triazolo[4,5-d]pyrimidin-7-yl)amino)ethoxy)ethoxy)ethyl)-2,2,6a,6b,9,9,12a-heptamethyl-10-(((2R,3R,5R,6R)-3,4,5-trihydroxy-6-(hydroxymethyl)tetrahydro-2H-pyran-2-yl)oxy)-1,3,4,5,6,6a,6b,7,8,8a,9,10,11,12,12a,12b,13,14b-octadecahydropicene-4a(2H)-carboxamide (Compound **C7**), 183 mg, yield is 83.2%, white solid. ^1^H-NMR (600 MHz, pyrdine-*d*5) *δ*: 10.15–10.11 (m, 1H), 7.49–7.47 (m, 1H), 5.84–5.80 (m, 4H), 5.49–5.4 (m, 4H), 4.90 (d, J = 7.7 Hz, 1H), 4.82 (s, 1H), 4.65 (s, 1H), 4.56–4.46 (m, 3H), 4.45 (s, 1H), 4.22 (dd, J = 9.5, 3.4 Hz, 1H), 4.16 (t, J = 6.1 Hz, 1H), 4.09–4.03 (m, 4H), 3.99–3.95 (m, 1H), 3.94–3.88 (m, 3H), 3.79–3.72 (m, 3H), 3.69–3.63 (m, 4H), 3.62 (s, 7H), 3.41 (dd, J = 11.8, 4.4 Hz, 1H), 3.29–3.17 (m, 2H), 3.06 (d, J = 10.3 Hz, 1H), 3.03–2.96 (m, 1H), 2.74–2.67 (m, 1H), 1.34 (s, H), 1.29 (s, 3H), 1.05 (t, J = 7.4 Hz, 3H), 1.00 (s, 3H), 0.96 (s, 3H), 0.94 (s, 3H), 0.93 (s, 3H), 0.92 (s, 3H). ^13^C-NMR (150 MHz, pyridine-*d*5) *δ* 178.24, 170.82, 154.84, 145.25, 124.99, 108.04, 89.14, 83.94, 77.24, 76.62, 75.97, 73.63, 72.88, 71.25, 71.06, 70.76, 70.69, 70.44, 62.88, 62.64, 62.27, 56.22, 50.14, 48.39, 47.23, 46.95, 42.62, 42.37, 41.52, 40.33, 40.23, 40.01, 39.22, 37.42, 34.96, 34.84, 34.12, 33.86, 33.67, 33.46, 31.35, 28.70, 28.28, 27.14, 26.58, 24.31, 24.20, 24.16, 23.91, 18.92, 17.85, 17.47, 16.04, 14.16. HRMS (ESI) calculated for C_56_H_91_N_7_NaO_13_S [M + Na]^+^: 1124.6293, found 1124.6281.

(4aS,6aS,6bR,10S,12aR)-N-(2-(2-(2-((3-((2S,3S,4S)-2,3-dihydroxy-4-(2-hydroxyethoxy)cyclopentyl)-5-(propylthio)-3H-[1,2,3]triazolo[4,5-d]pyrimidin-7-yl)amino)ethoxy)ethoxy)ethyl)-2,2,6a,6b,9,9,12a-heptamethyl-10-(((2S,3R,5R)-3,4,5-trihydroxytetrahydro-2H-pyran-2-yl)oxy)-1,3,4,5,6,6a,6b,7,8,8a,9,10,11,12,12a,12b,13,14b-octadecahydropicene-4a(2H)-carboxamide (Compound **C8**), 175 mg, yield is 81.8%, white solid. ^1^H-NMR (600 MHz, pyridine-*d*5) *δ*: 10.16–10.11 (m, 1H), 7.49–7.47 (m, 1H), 5.50–5.47 (m, 2H), 5.45–5.43 (m, 1H), 4.87 (d, J = 7.5 Hz, 1H), 4.82–4.80 (m, 1H), 4.47–4.40 (m, 2H), 4.30–4.25 (m, 1H), 4.22 (t, J = 8.8 Hz, 1H), 4.07–4.02 (m, 6H), 3.98–3.95 (m, 1H), 3.93–3.89 (m, 3H), 3.84–3.79 (m, 1H), 3.78–3.73 (m, 3H), 3.69–3.63 (m, 5H), 3.62 (s, 3H), 3.38 (dd, J = 11.8, 4.4 Hz, 1H), 3.28–3.17 (m, 2H), 3.08–3.03 (m, 1H), 3.03–2.96 (m, 1H), 2.75–2.66 (m, 1H), 1.34 (s, 3H), 1.28 (s, 3H), 1.04 (t, J = 7.3 Hz, 3H), 1.03 (s, 3H), 0.97 (s, 3H), 0.94–0.93 (m, 9H). ^13^C-NMR (150 MHz, pyridine-*d*5) *δ* 178.24, 170.82, 154.84, 145.20, 124.99, 108.22, 89.07, 83.95, 79.14, 76.64, 76.04, 75.96, 72.89, 71.72, 71.25, 71.07, 70.69, 70.45, 67.63, 62.65, 62.27, 56.24, 48.43, 47.20, 46.95, 42.61, 42.37, 41.51, 40.33, 40.24, 40.06, 39.25, 37.46, 34.97, 34.83, 34.11, 33.86, 33.67, 33.45, 31.35, 28.65, 28.28, 27.22, 26.53, 24.33, 24.21, 23.91, 18.94, 17.86, 17.45, 16.06, 14.17. HRMS (ESI) calculated for C_55_H_89_N_7_NaO_12_S [M + Na]^+^: 1094.6188, found 1094.6198.

(4aS,6aS,6bR,10S,12aR)-N-(2-(2-(2-((3-((2S,3S,4S)-2,3-dihydroxy-4-(2-hydroxyethoxy)cyclopentyl)-5-(propylthio)-3H-[1,2,3]triazolo[4,5-d]pyrimidin-7-yl)amino)ethoxy)ethoxy)ethyl)-2,2,6a,6b,9,9,12a-heptamethyl-10-(((2S,3S,4R,5R)-3,4,5-trihydroxytetrahydro-2H-pyran-2-yl)oxy)-1,3,4,5,6,6a,6b,7,8,8a,9,10,11,12,12a,12b,13,14b-octadecahydropicene-4a(2H)-carboxamide (Compound **C9**), 189 mg, yield is 88.3%, white solid. ^1^H-NMR (600 MHz, pyridine-*d*5) *δ*: 10.15–10.10 (m, 1H), 7.52–7.47 (m, 1H), 5.87–5.80 (m, 1H), 5.79–5.71 (s, 2H), 5.50–5.43 (m, 3H), 4.83–4.78 (m, 2H), 4.51 (t, J = 7.9 Hz, 1H), 4.43–4.40 (m, 1H), 4.40 (s, 1H), 4.38–4.34 (m, 1H), 4.22 (dd, J = 8.8, 3.5 Hz, 1H), 4.07–4.03 (m, 4H), 3.99–3.95 (m, 1H), 3.94–3.89 (m, 3H), 3.86 (dd, J = 12.3, 1.7 Hz, 1H), 3.78–3.73 (m, 3H), 3.70–3.64 (m, 4H), 3.37 (dd, J = 11.8, 4.4 Hz, 1H), 3.29–3.16 (m, 2H), 3.08–3.03 (m, 1H), 3.03–2.96 (m, 1H), 2.74–2.67 (m, 1H), 1.31 (s, 3H), 1.27 (s, 3H), 1.05 (t, J = 7.4 Hz, 3H), 0.99 (s, 3H), 0.97 (s, 3H), 0.95–0.93 (m, 9H). ^13^C-NMR (150 MHz, pyridine-*d*5) *δ*: 178.25, 170.82, 154.84, 145.20, 124.99, 108.07, 89.12, 83.94, 76.63, 75.98, 75.13, 73.34, 72.88, 71.25, 71.07, 70.69, 70.44, 70.06, 67.21, 62.65, 62.28, 56.25, 50.15, 48.42, 47.21, 46.96, 42.60, 42.36, 41.52, 40.33, 40.24, 40.05, 39.25, 37.46, 34.97, 34.84, 34.12, 33.86, 33.67, 33.45, 31.35, 28.68, 28.27, 27.13, 26.53, 24.33, 24.20, 24.17, 23.91, 18.94, 17.86, 17.41, 16.06, 14.17. HRMS (ESI) calculated for C_55_H_89_N_7_NaO_12_S [M + Na]^+^: 1094.6188, found 1094.6193.

(4aS,6aS,6bR,10S,12aR)-N-(2-(2-(2-((3-((2S,3S,4S)-2,3-dihydroxy-4-(2-hydroxyethoxy)cyclopentyl)-5-(propylthio)-3H-[1,2,3]triazolo[4,5-d]pyrimidin-7-yl)amino)ethoxy)ethoxy)ethyl)-2,2,6a,6b,9,9,12a-heptamethyl-10-(((2R,3R,4R,5R,6S)-3,4,5-trihydroxy-6-methyltetrahydro-2H-pyran-2-yl)oxy)-1,3,4,5,6,6a,6b,7,8,8a,9,10,11,12,12a,12b,13,14b-octadecahydropicene-4a(2H)-carboxamide (Compound **C10**), 164 mg, yield is 75.6%, white solid. ^1^H-NMR (600 MHz, pyridine-*d*5) *δ*: 10.17–10.11 (m, 1H), 7.49 (t, J = 5.5 Hz, 1H), 5.82 (d, J = 8.9 Hz, 1H), 5.48 (s, 1H), 5.46 (d, J = 3.6 Hz, 1H), 5.36 (s, 1H), 4.82–4.79 (m, 1H), 4.65–4.61 (m, 1H), 4.55–4.51 (m, 1H), 4.44–4.39 (m, 1H), 4.38–4.35 (m, 2H), 4.06 (t, J = 5.4 Hz, 3H), 3.99–3.95 (m, 1H), 3.93–3.89 (m, 3H), 3.79–3.73 (m, 3H), 3.70–3.64 (m, 4H), 3.62 (s, 3H), 3.29–3.23 (m, 1H), 3.23–3.15 (m, 2H), 3.06 (d, J = 10.0 Hz, 1H), 3.03–2.97 (m, 1H), 2.75–2.67 (m, 1H), 1.71 (s, 3H), 1.26 (s, 3H), 1.05 (t, J = 7.3 Hz, 3H), 0.97 (s, 3H), 0.94 (d, J = 4.0 Hz, 12H), 0.83 (s, 3H). ^13^C-NMR (150 MHz, pyridine-*d*5) *δ*: 178.25, 170.82, 154.84, 145.20, 124.99, 108.07, 89.12, 83.94, 76.63, 75.98, 75.13, 73.34, 72.88, 71.25, 71.07, 70.69, 70.44, 70.06, 67.21, 62.65, 62.28, 56.25, 50.15, 48.42, 47.21, 46.96, 42.60, 42.36, 41.52, 40.33, 40.24, 40.05, 39.25, 37.46, 34.97, 34.84, 34.12, 33.86, 33.67, 33.45, 31.35, 28.68, 28.27, 27.13, 26.53, 24.33, 24.20, 24.17, 23.91, 18.94, 17.86, 17.41, 16.06, 14.17. HRMS (ESI) calculated for C_56_H_91_N_7_NaO_12_S [M + Na]^+^: 1108.6344, found 1108.6370.

### 3.2. Biological Assay

#### 3.2.1. Cell Culture and Treatment

HUVECs were provided by Genmei Technology 210 Co., Ltd. (Wuhan, China), which were grown in endothelial cell culture medium (No. 1001, Sciencell) containing 5% fetal bovine serum (FBS), 1% endothelial cell growth supplement, and 1% penicillin/streptomycin solution in 5% CO_2_ at 37 °C. Compounds were dissolved in DMSO, and the mother liquor was then diluted with culture medium for the experiments. HUVECs were administrated with the compound for 24 h and then added to ox-LDL (50 μg/mL) for 2.5 h.

#### 3.2.2. Cell Viability Assays

Cell viability was detected with the cell counting kit-8 (CCK-8) assay. HUVECs in the logarithmic growth phase were collected and adjusted to a density of 1 × 10^5^ cells/mL. The cells were seeded into 96-well plates and cultured at 37 °C with 5% CO_2_ until reaching 80–90% confluence. Cells were then treated according to the following groups: Control group: incubated with serum-free endothelial cell culture medium only. Model group: cultured with serum-free medium for 24 h, followed by incubation with 50 μg/mL high-ox-LDL prepared in serum-free medium for 2.5 h. Drug treatment groups: pretreated with various concentrations of compounds for 24 h, followed by incubation with 50 μg/mL high-ox-LDL for 2.5 h. After the above treatments, the culture medium was replaced with serum-free medium containing 10% CCK-8 solution (WJ30025). The plates were incubated at 37 °C for 2–3 h. Absorbance at 450 nm was measured by a microplate reader (357-913409, Thermo Fisher Scientific, Waltham, MA, USA).

#### 3.2.3. Surface Plasmon Resonance (SPR)

Interactions and kinetic constants between small molecules and proteins were detected through a Biacore system using the amino coupling method. A CM 5 chip was treated with sulpho-NHS and EDC (2.7 mM KCl, 137 mM NaCl, 0.05% (*v*/*v*) p20, pH 7.4 buffer). Then, recombinant human HSP90β protein was immobilized on one channel of the chip, and another channel was used as the reference channel. HSP90β coupling conditions were as follows: 10 μg/mL, pH 4.0 sodium acetate buffer, followed by sealing with 1 M ethanolamine (pH 8.0). The compounds were dissolved in 100% DMSO to prepare a 10 mM stock solution, which was then diluted with loading buffer containing 5% DMSO to concentrations of 7.8, 3.9, 1.95, 0.98, and 0.49 μM for sample loading. Compounds were added in 100% DMSO at 10 mM and then diluted with running buffer containing 5% DMSO to 7.8, 3.9, 1.95, 0.98, and 0.49 μM for loading. Data were analyzed using Biacore S200 evaluation software (Biacore S200 Evaluation Software 1.1.1).

#### 3.2.4. Molecular Docking (MD)

Based on the crystal structure of HSP90β (PDB ID: 3NMQ) and P2Y12 (PDB ID: 4NTJ), we docked **C5** to HSP90β and **C5** to P2Y12 by using MOE software (MOE 2019.0102). In order to explore the potential binding of **C5** with HSP90β (PDB ID: 3NMQ) and P2Y12 (PDB ID: 4NTJ), molecular modeling was conducted in MOE software (MOE 2019.0102) using the induced-fit docking protocol. Ligand **C5** underwent “energy minimization” prior to docking to eliminate any bond length and angle deviations. Binding affinity (s value) in MOE was used to evaluate the interaction between HSP90β or P2Y12 and **C5**. The score (binding affinity) was obtained based on virtual calculations of various interactions between the ligand and the target receptor. The docking diagram of **C5** and HSP90β or P2Y12 were drawn by PyMOL 2.5.2.

#### 3.2.5. Microscale Thermophoresis (MST)

Binding affinities between CE/Ticagrelor and HSP90β were measured at 25 °C in PBST binding buffer containing 5% DMSO by MST using an MONOLITH NT.115 system (NanoTemper Technologies, Munich, Germany). Briefly, 10 μL of HSP90β (0.1 μM) was mixed with 10 μL of twofold serially diluted ligands (250 μM) to reach a final protein concentration of 50 nM. Data were plotted using MO.Affinity Analysis v2.3 (NanoTemper Technologies, Munich, Germany).

#### 3.2.6. Statistical Analysis

GraphPad Prism 8.0.2 was used for statistical analysis, and experimental data are showed as mean ± standard deviation (mean ± SD). The significance of differences among groups was tested through one-way analysis of variance with post hoc Turkey test. *p* < 0.05 denoted statistical significance.

## 4. Conclusions

Atherosclerosis (AS) serves as the primary pathological basis for cardiovascular disease-related deaths globally, and its incidence is showing a younger trend. Previous studies have confirmed that Calenduloside E (CE), a pentacyclic triterpenoid from natural products, possesses anti-atherosclerotic activity. However, its clinical translation potential remains to be explored due to limitations such as insufficient activity and a narrow therapeutic index.

Herein, we report ten CE derivatives synthesized via a molecular hybridization strategy. These derivatives were constructed by linking a CE fragment with a ticagrelor moiety through PEG chains, with different sugar groups introduced simultaneously. Through cytotoxicity screening and ox-LDL-induced HUVEC injury model, derivative **C5** was identified as the one with the optimal activity. Compared with CE, the EC_50_ value of **C5** was reduced to 1.44 μM, which successfully expanded the therapeutic index of CE, providing a structural basis for improving the safety of clinical medication. Molecular docking verified that derivative **C5**, similar to the parent compound CE, could target HSP90β by forming specific hydrogen bonds with the key amino acid residue Asp93. Further SPR experiments demonstrated that the binding affinities of **C5** and CE to HSP90β were 1.73 μM and 1.72 μM, respectively. This suggests that **C5** may exert activity against ox-LDL-induced HUVEC injury by targeting HSP90β, consistent with the mechanism of CE. To clarify the regulatory role of the ticagrelor active fragment in targeting HSP90β, we determined the binding abilities of **C5** and ticagrelor monomer to HSP90β using MST. The dissociation constants (Kd) of **C5** and ticagrelor were 14.87 μM and 1.65 mM, respectively.. The dissociation constants (Kd) were 14.87 μM and 1.65 mM, respectively. The binding affinity of **C5** to HSP90β was approximately 111 times that of ticagrelor, indicating that the ticagrelor fragment does not enhance activity by directly strengthening binding to HSP90β, and its function may be related to the regulation of other targets. To explore additional potential targets of **C5** in endothelial protection, we performed target prediction using the SwissTargetPrediction online database and verified the result via molecular docking. P2Y12 was identified as the most promising candidate target. The ticagrelor-derived active fragment within **C5** formed hydrogen bond with Lys280, a key amino acid residue of P2Y12, thereby providing molecular evidence that **C5** exerts anti-atherosclerotic activity via P2Y12 targeting. The ticagrelor active fragment in **C5** could form a hydrogen bond with the key amino acid residue Lys280 of P2Y12, providing molecular evidence that **C5** exert anti-atherosclerosis activity by targeting P2Y12.

In summary, this study obtained CE derivative **C5** with enhanced anti-atherosclerosis activity through rational structural design and systematic activity screening. This compound not only reduces the effective concentration of the parent CE but also provides important experimental basis for reducing dosage in subsequent clinical applications and minimizing potential toxic side effects caused by high-dose exposure. Meanwhile, it reveals another potential target of **C5**, laying a foundation for further structural optimization and mechanistic research of this type of compounds.

## Figures and Tables

**Table 1:** Identified targets of compound **C5** by SwissTargetPrediction. (Top 13).

Target	Common Name	Uniprot ID	ChEMBL ID	Target Class
Purinergic receptor P2Y12	P2RY12	Q9H244	CHEMBL2001	Family A G protein-coupled receptor
Phosphodiesterase 8B	PDE8B	O95263	CHEMBL4408	Phosphodiesterase
Butyrylcholinesterase	BCHE	P06276	CHEMBL1914	Hydrolase
Acetylcholinesterase	ACHE	P22303	CHEMBL220	Hydrolase
Serine/threonine protein kinase mTOR	MTOR	P42345	CHEMBL2842	Kinase
PI3-kinase p110-gamma subunit	PIK3CG	P48736	CHEMBL3267	Enzyme
PI3-kinase p110-alpha subunit	PIK3CA	P42336	CHEMBL4005	Enzyme
DNA (cytosine-5)-methyltransferase 1	DNMT1	P26358	CHEMBL1993	Writer
DNA (cytosine-5)-methyltransferase 3B	DNMT3B	Q9UBC3	CHEMBL6095	Reader
Adenosine deaminase	ADA	P00813	CHEMBL1910	Hydrolase
6-O-methylguanine-DNA methyltransferase	MGMT	P16455	CHEMBL2864	Enzyme
PI3-kinase p110-delta subunit	PIK3CD	O00329	CHEMBL3130	Enzyme
PI3-kinase p110-beta subunit	PIK3CB	P42338	CHEMBL3145	Enzyme

## Data Availability

The original contributions presented in this study are included in the article/Supplementary Materials. Further inquiries can be directed to the corresponding authors.

## Supplementary Materials

The following supporting information can be downloaded at: https://www.mdpi.com/article/10.3390/ph19010090/s1, Figure S1. ^1^H NMR spectra of compound **C1**. Figure S2. ^13^C-NMR spectra of compound **C1**. Figure S3. HRMS spectra of compound **C1**. Figure S4. ^1^H NMR spectra of compound **C2**. Figure S5. ^13^C-NMR spectra of compound **C2**. Figure S6. HRMS spectra of compound **C2** Figure S7. ^1^H NMR spectra of compound **C3**. Figure S8. ^13^C-NMR spectra of compound **C3**. Figure S9. HRMS spectra of compound **C3**. Figure S10. ^1^H NMR spectra of compound **C4**. Figure S11. ^13^C-NMR spectra of compound **C4**. Figure S12. HRMS spectra of compound **C4**. Figure S13. ^1^H NMR spectra of compound **C5**. Figure S14. ^13^C-NMR spectra of compound **C5**. Figure S15. HRMS spectra of compound **C5**. Figure S16. ^1^H NMR spectra of compound **C6**. Figure S17. ^13^C-NMR spectra of compound **C6**. Figure S18. HRMS spectra of compound **C6**. Figure S19. ^1^H NMR spectra of compound **C7**. Figure S20. ^13^C-NMR spectra of compound **C7**. Figure S21. HRMS spectra of compound **C7**. Figure S22. ^1^H NMR spectra of compound **C8**. Figure S23. ^13^C-NMR spectra of compound **C8**. Figure S24. HRMS spectra of compound **C8**. Figure S25. ^1^H NMR spectra of compound **C9**. Figure S26. ^13^C-NMR spectra of compound **C9**. Figure S27. HRMS spectra of compound **C9**. Figure S28. ^1^H NMR spectra of compound **C10**. Figure S29. ^13^C-NMR spectra of compound C10. Figure S30. HRMS spectra of compound **C10**. Figure S31. Protective effects of compound **C5** against ox-LDL-induced damage in HUVECs cells. (A) Protective effects of compound **C5**, CE, ticagrelor and mixture of CE and ticagrelor (1:1) at 3.125 μM. Data are presented as mean ± SD of each group (*n* = 3). ^###^ *p* < 0.001 vs Control group, *** *p* < 0.001 vs Model group. (B) The toxicity assessment of mixture of CE and ticagrelor (1:1) at 3.125 μM. Data are presented as mean ± SD of each group (*n* = 3). *** *p* < 0.001 vs Control group.

## Abbreviations

The following abbreviations are used in this manuscript:

ACS	Acute coronary syndrome
ApoE^−/−^	ApoE knockout
AS	Atherosclerosis
cAMP	Cyclic adenosine monophosphate
CCK-8	Cell counting kit-8
CE	Calenduloside E
CPTP	Cyclopentyl–triazolopyrimidine
DCM	Dichloromethane
DMSO	Dimethyl sulfoxide
EA	Ethyl acetate
EDCI	1-Ethyl-3-(3-dimethyllaminopropyl) carbodiimide hydrochloride
eNOS	Endothelial nitric oxide synthase
HOBT	1-Hydroxybenzotriazole
HSP90	Heat shock protein 90
HUVECs	Human umbilical vein endothelial cells
IL–6	Interleukin-6
ka	Association rate constant
K_D_	Equilibrium dissociation constant
kd	Dissociation rate constant
KLF2	Kruppel-like factor 2
LTCC	L-type calcium channels
CH_3_OH	Methanol
MOE	Molecular operating environment
MST	Microscale thermophoresis
NF-κB	Nuclear factor-κB
NO	Nitric oxide
ox-LDL	Oxidized low-density lipoprotein
Pd/C	10% palladium on carbon
PE	Petroleum ether
SPR	Surface plasmon resonance
TBAB	Tetrabutylammonium bromide
TLC	Thin-layer chromatography
TMSOTf	Trimethylsilyl trifluoromethanesulfonate
TNF-α	Tumor necrosis factor-α
VSMCs	Vascular smooth muscle cells
