# Supplementary material for: Discovery and Evaluation of Novel Calenduloside E Derivatives Targeting HSP90β in Ox-LDL-Induced HUVECs Injury
Source: Pharmaceuticals (Basel). 2026 Jan 2;19(1):90. doi: 10.3390/ph19010090 (PMC12845407; doi:10.3390/ph19010090)
Supplement: Supplementary file 1 [file pharmaceuticals-19-00090-s001.zip › pharmaceuticals-4033626-supplementary.pdf]

Supplementary information

Discovery and Evalucation of Novel Calenduloside E Derivatives Targeting HSP90β in ox-LDL-induced HUVECs injury

Fang Han 1, †, Huiqi Fang 1, † , Guangyu Li 1 , Di Deng 1 , Guibo Sun 1, * and Yu Tian 1, *

1 State Key Laboratory for Quality Ensurance and Sustainable Use of Dao-di Herbs, Institute of Medicinal Plant Development, Chinese Academy of Medical Sciences and Peking Union Medical College, Beijing 100193, China.

* Correspondence: sunguibo@126.com (G. Sun), ytian@implad.ac.cn (Y. Tian).

† These authors contributed equally to this work.

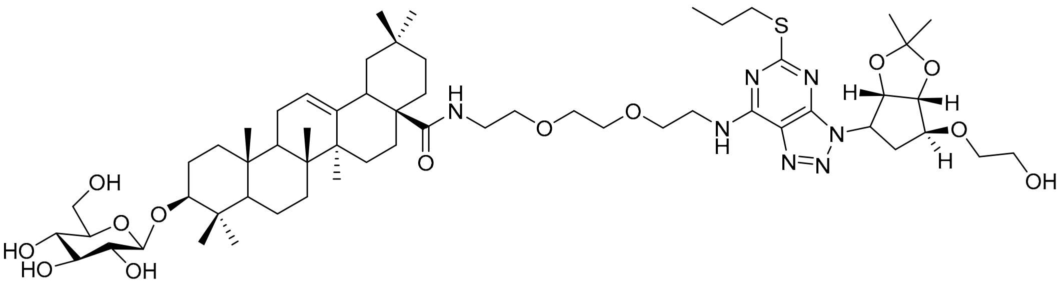

Compound C1

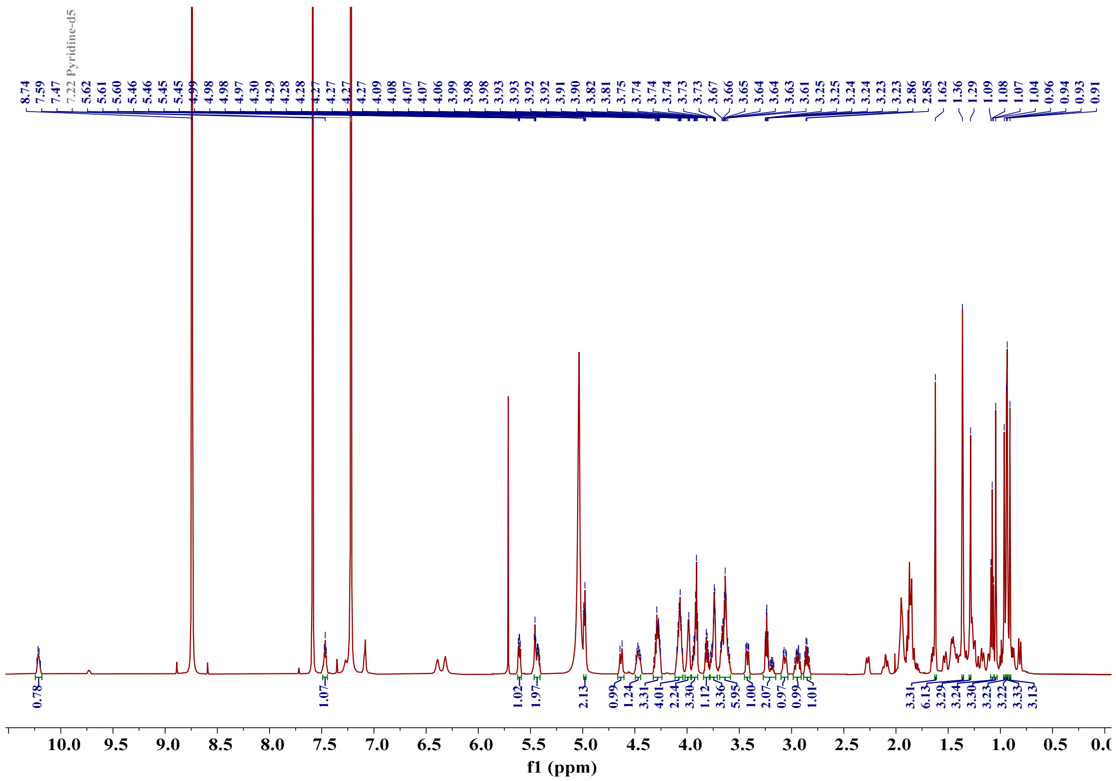

Figure S1. ^{1}H NMR spectra of compound **C1**.

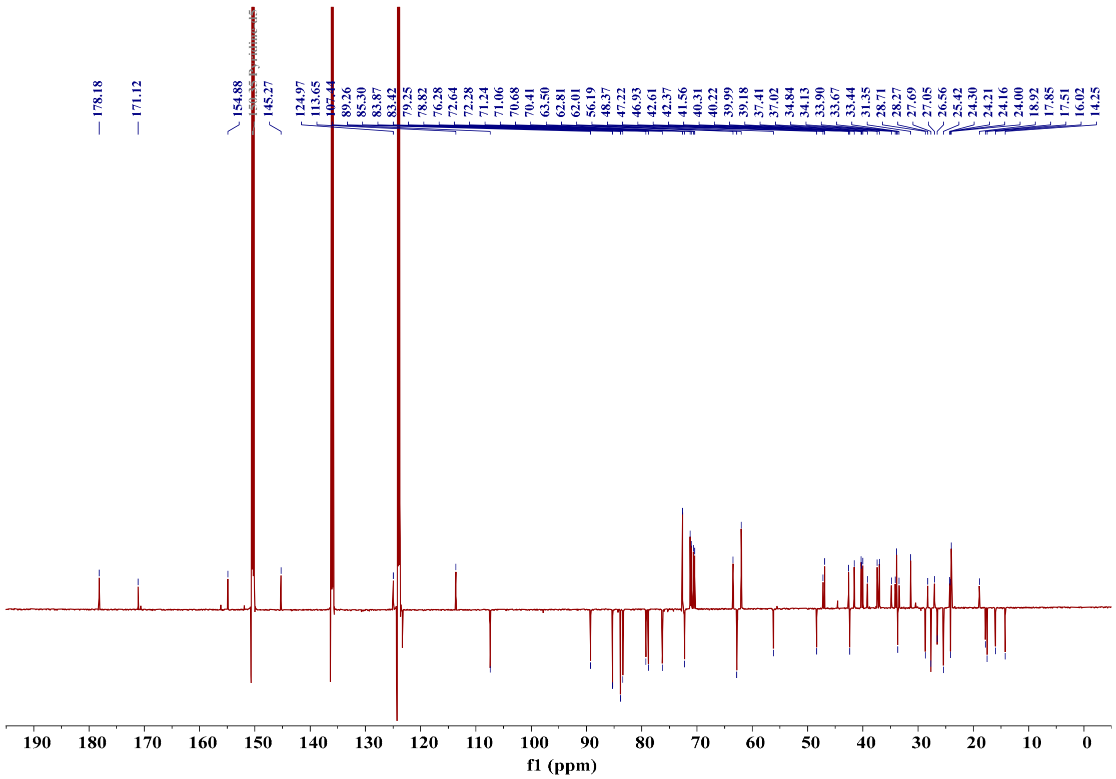

Figure S2. ^{13}C-NMR spectra of compound **C1**.

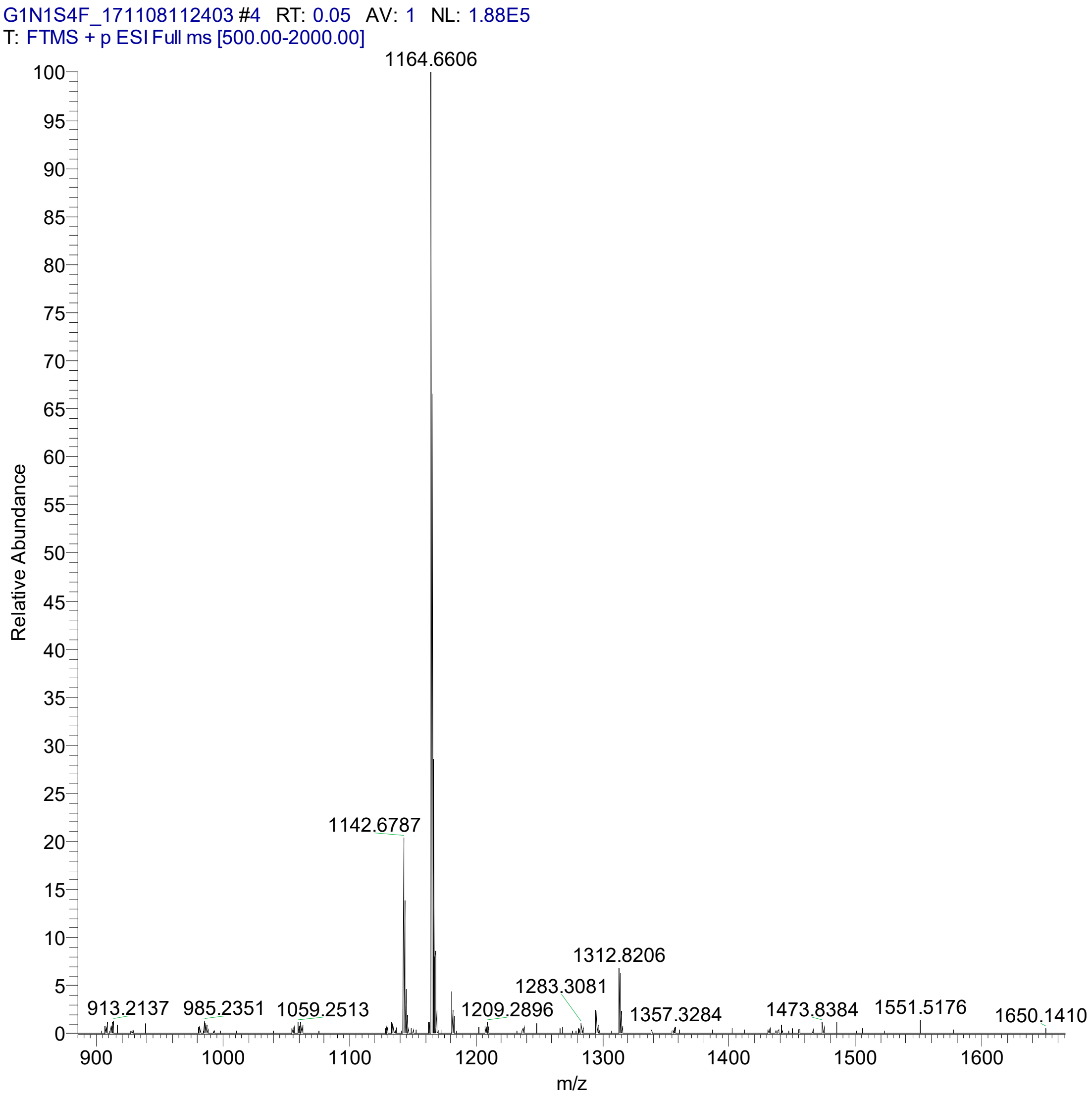

Figure S3. HRMS spectra of compound **C1**.

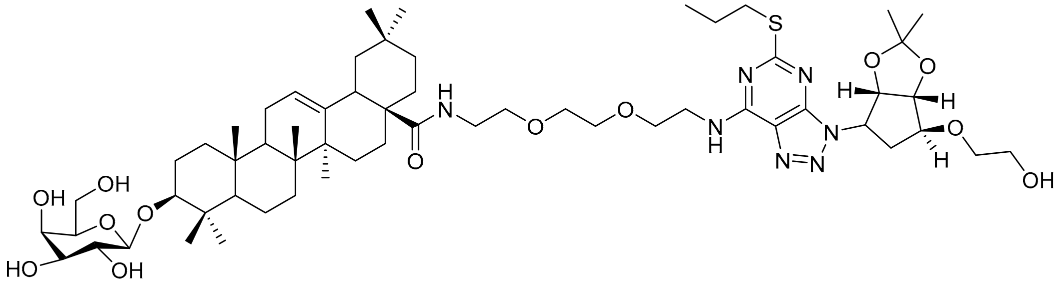

Compound C2

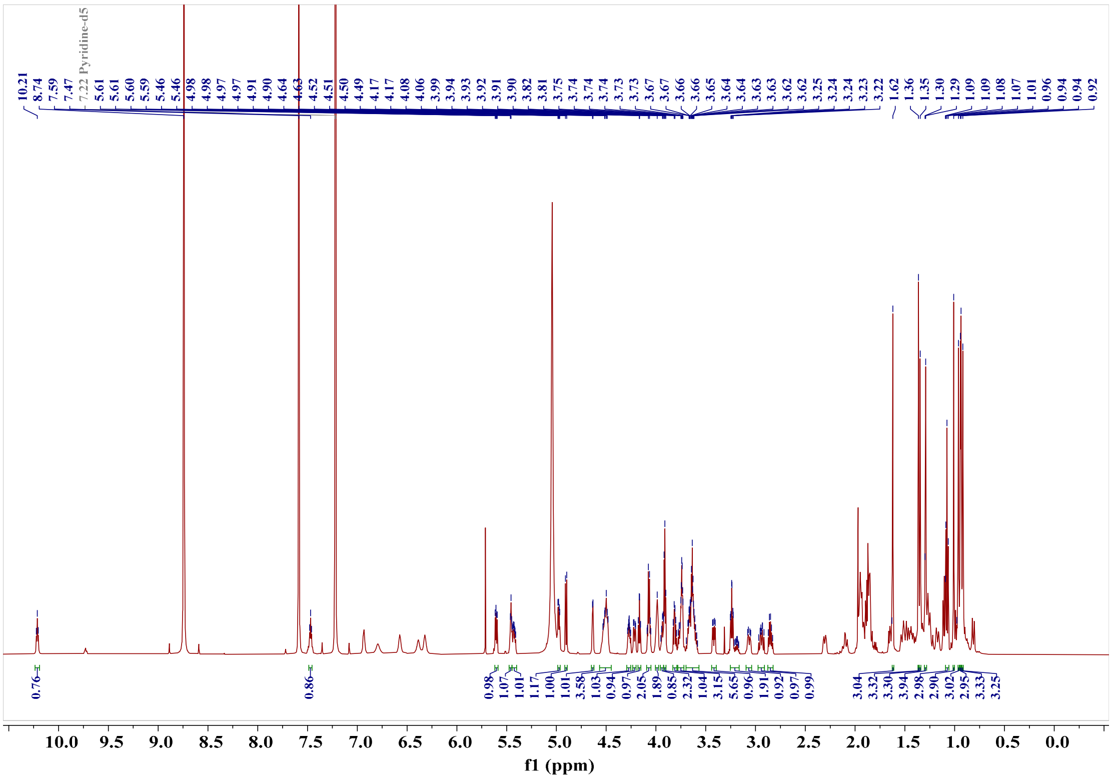

Figure S4. ^{1}H NMR spectra of compound **C2**.

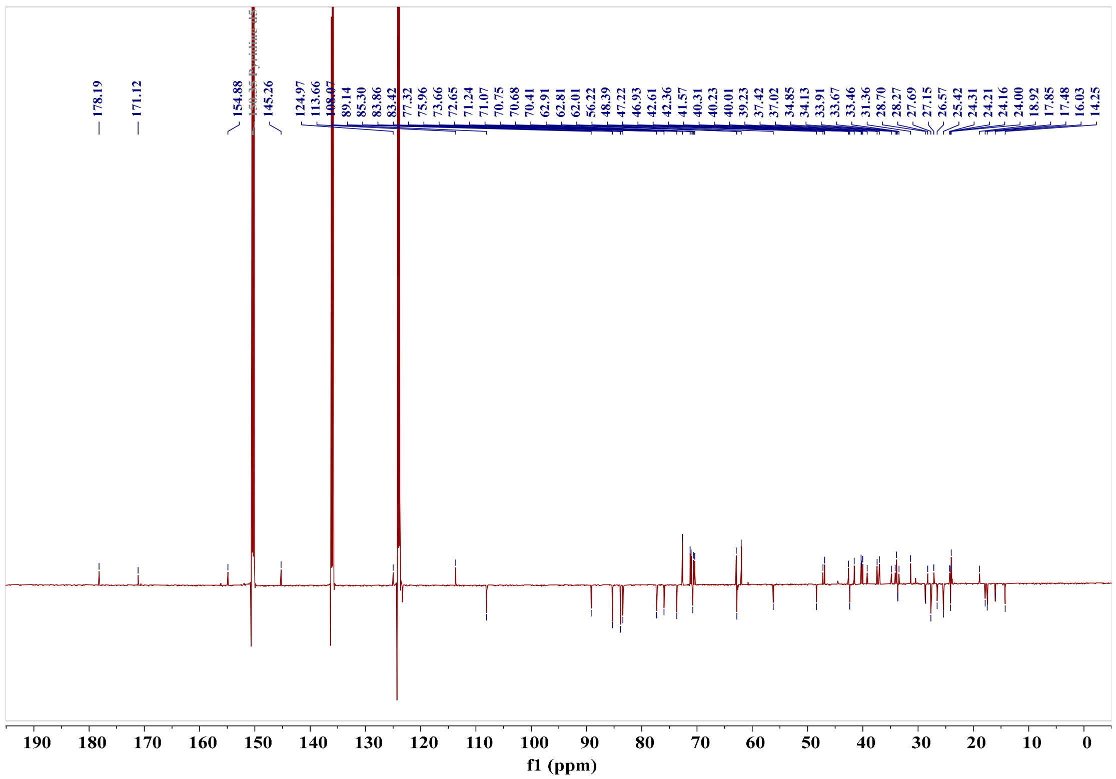

Figure S5. ^{13}C-NMR spectra of compound **C2**.

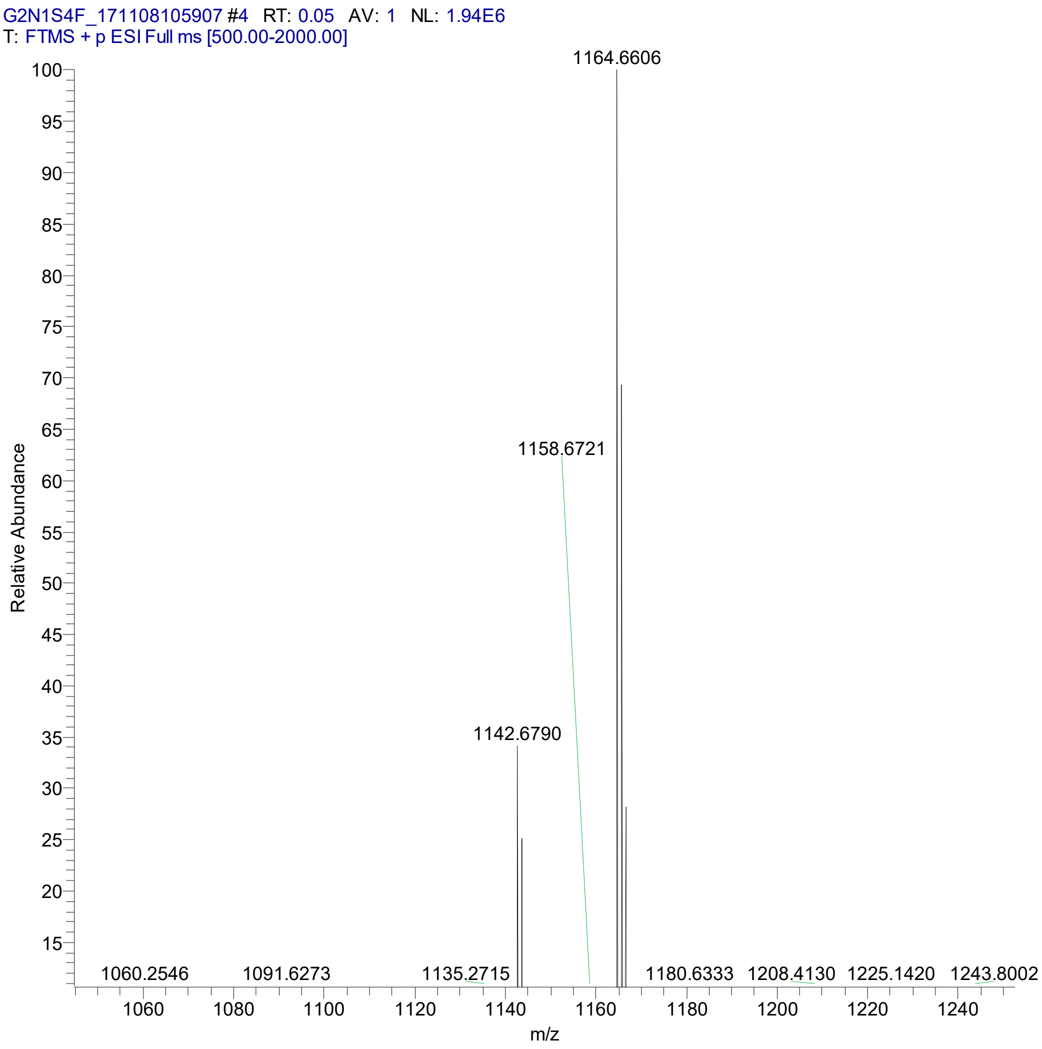

Figure S6. HRMS spectra of compound **C2**

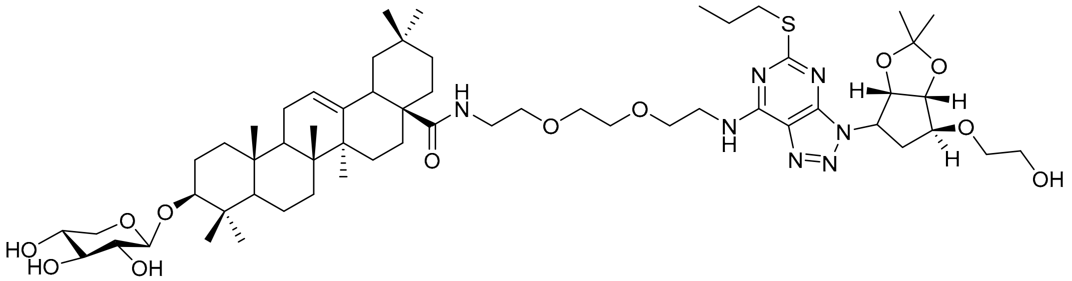

Compound C3

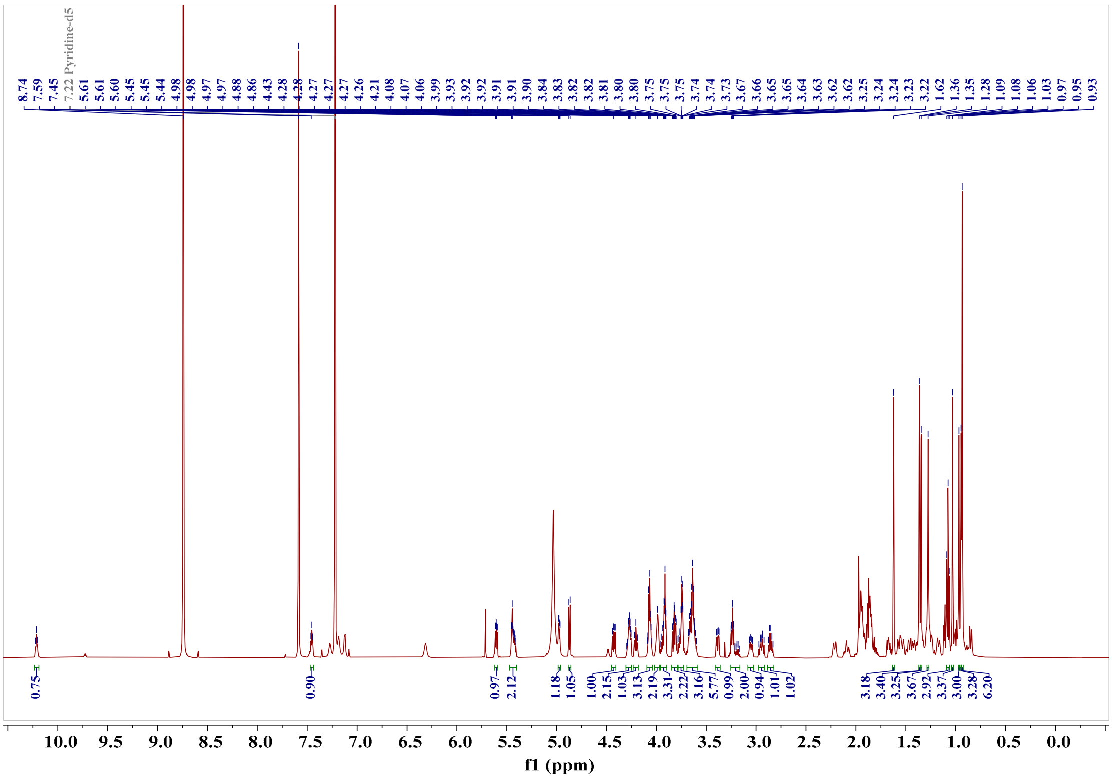

Figure S7. ^{1}H NMR spectra of compound **C3**.

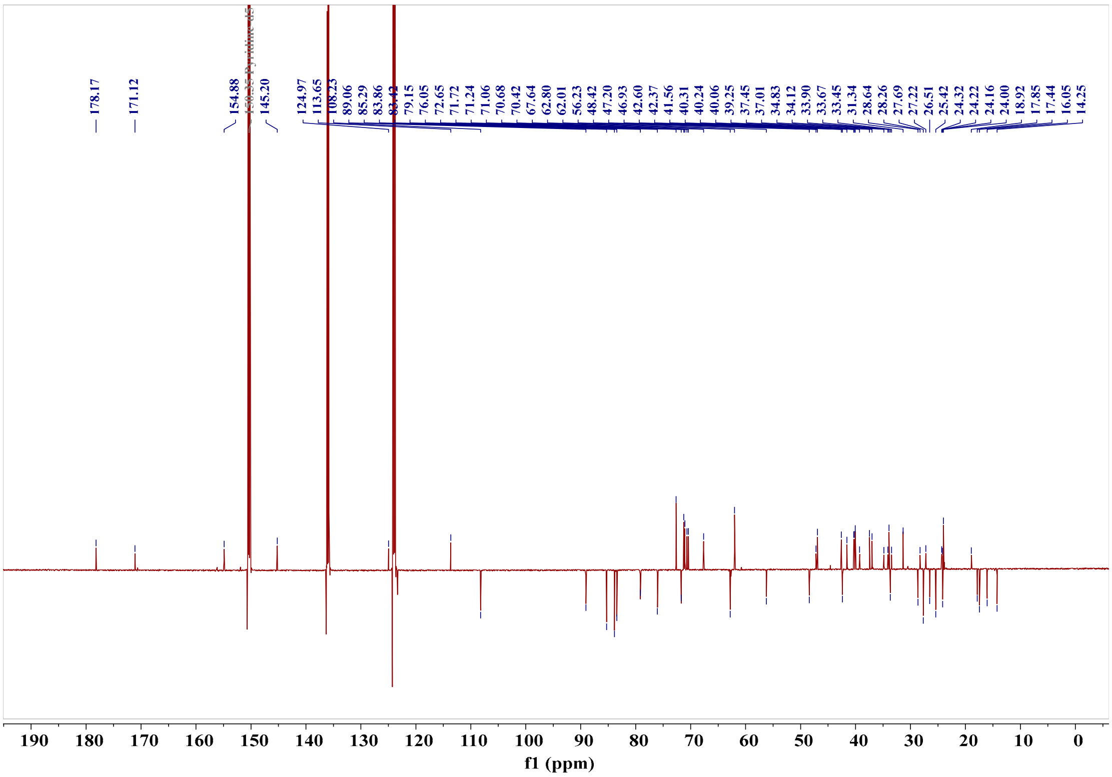

Figure S8. ^{13}C-NMR spectra of compound **C3**.

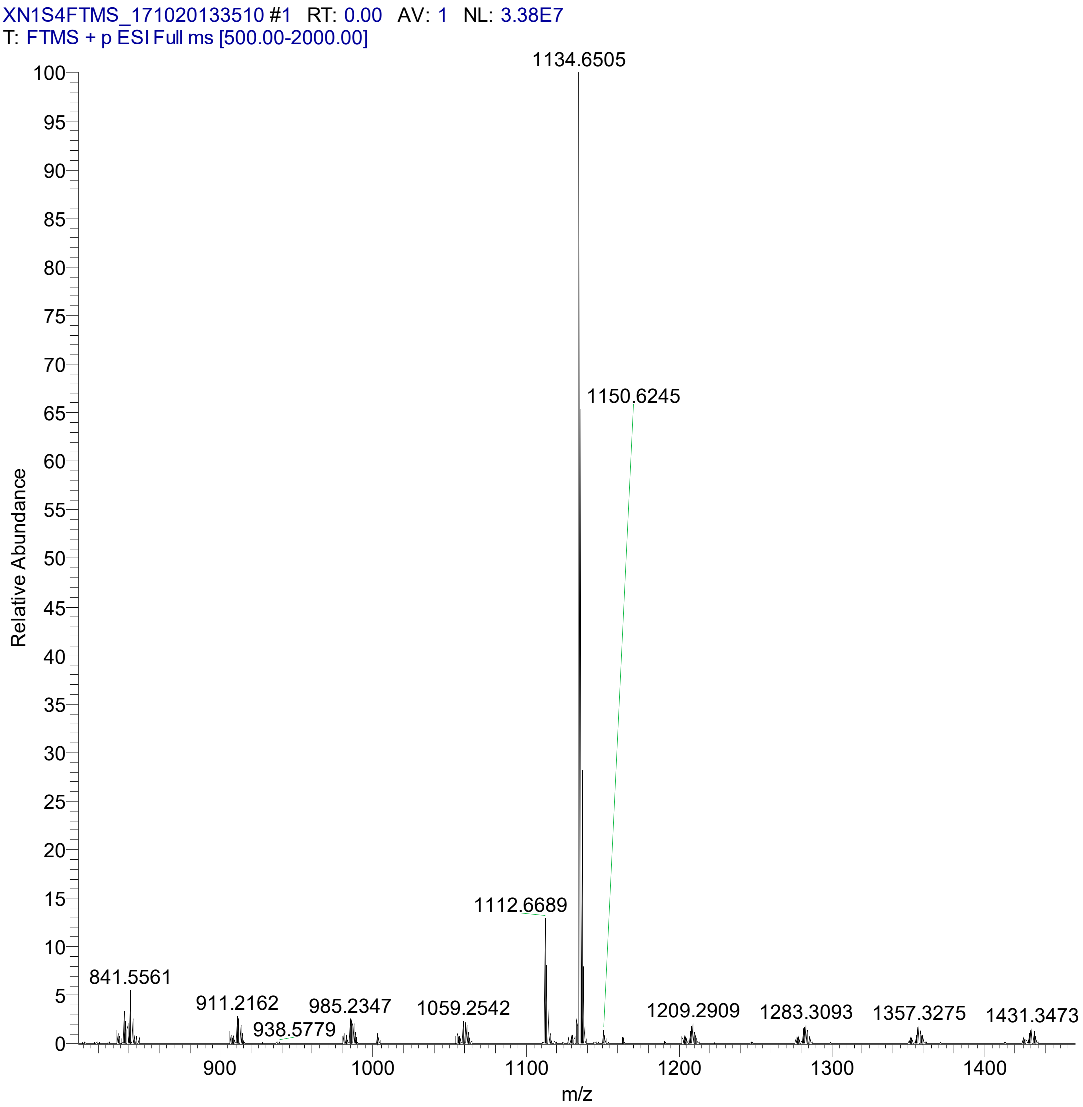

Figure S9. HRMS spectra of compound **C3**.

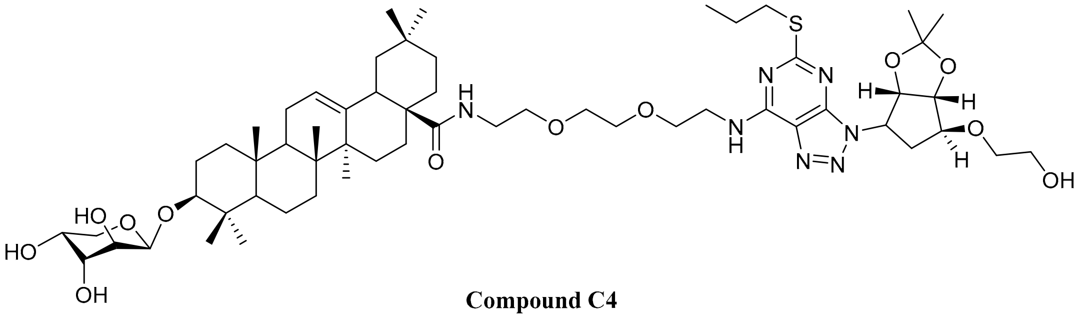

Compound C4

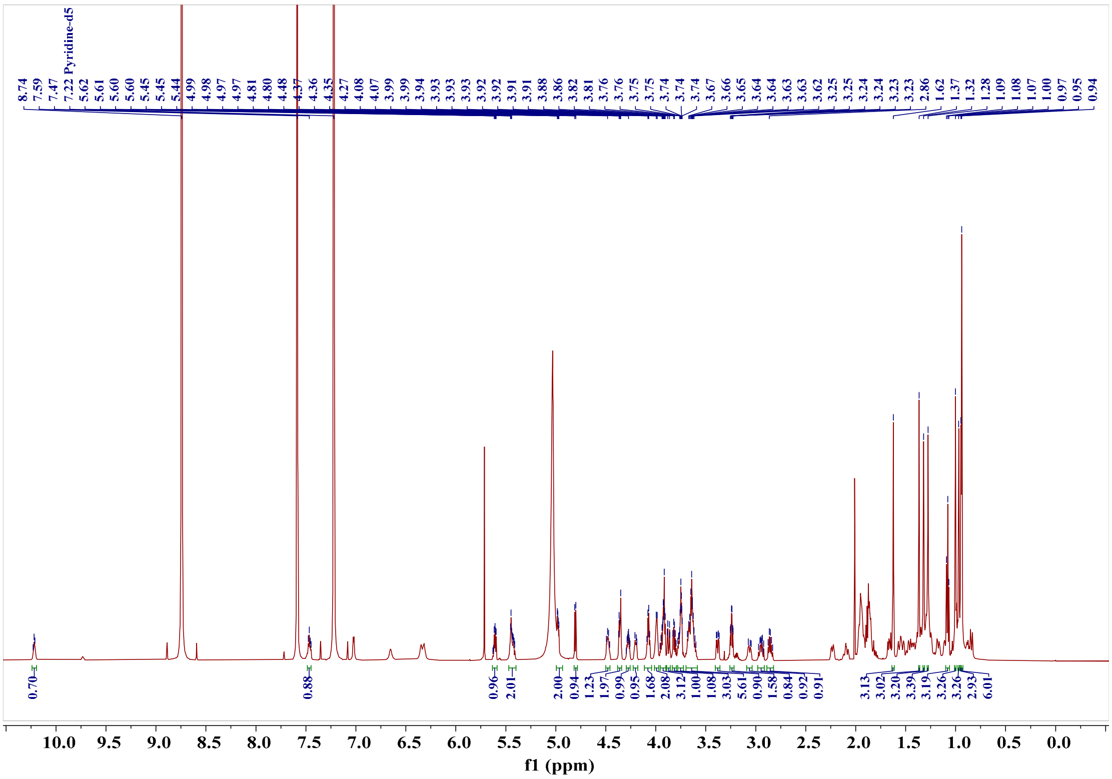

Figure S10. ^{1}H NMR spectra of compound **C4**.

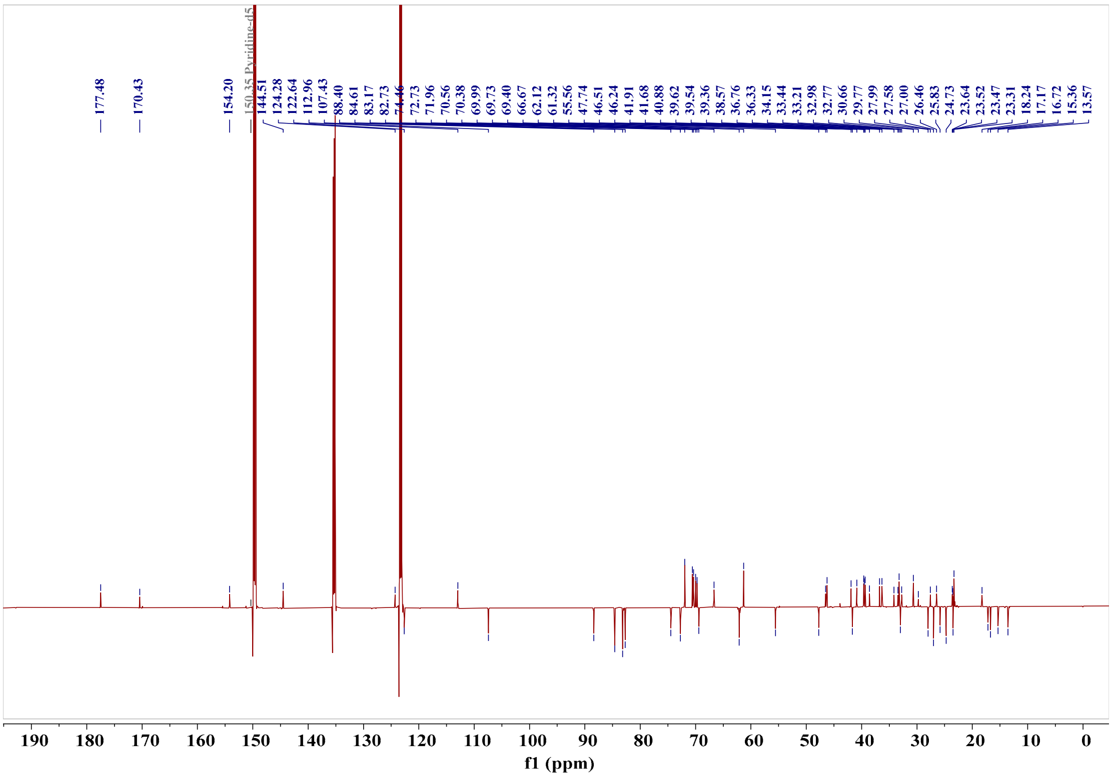

Figure S11. ^{13}C-NMR spectra of compound **C4**.

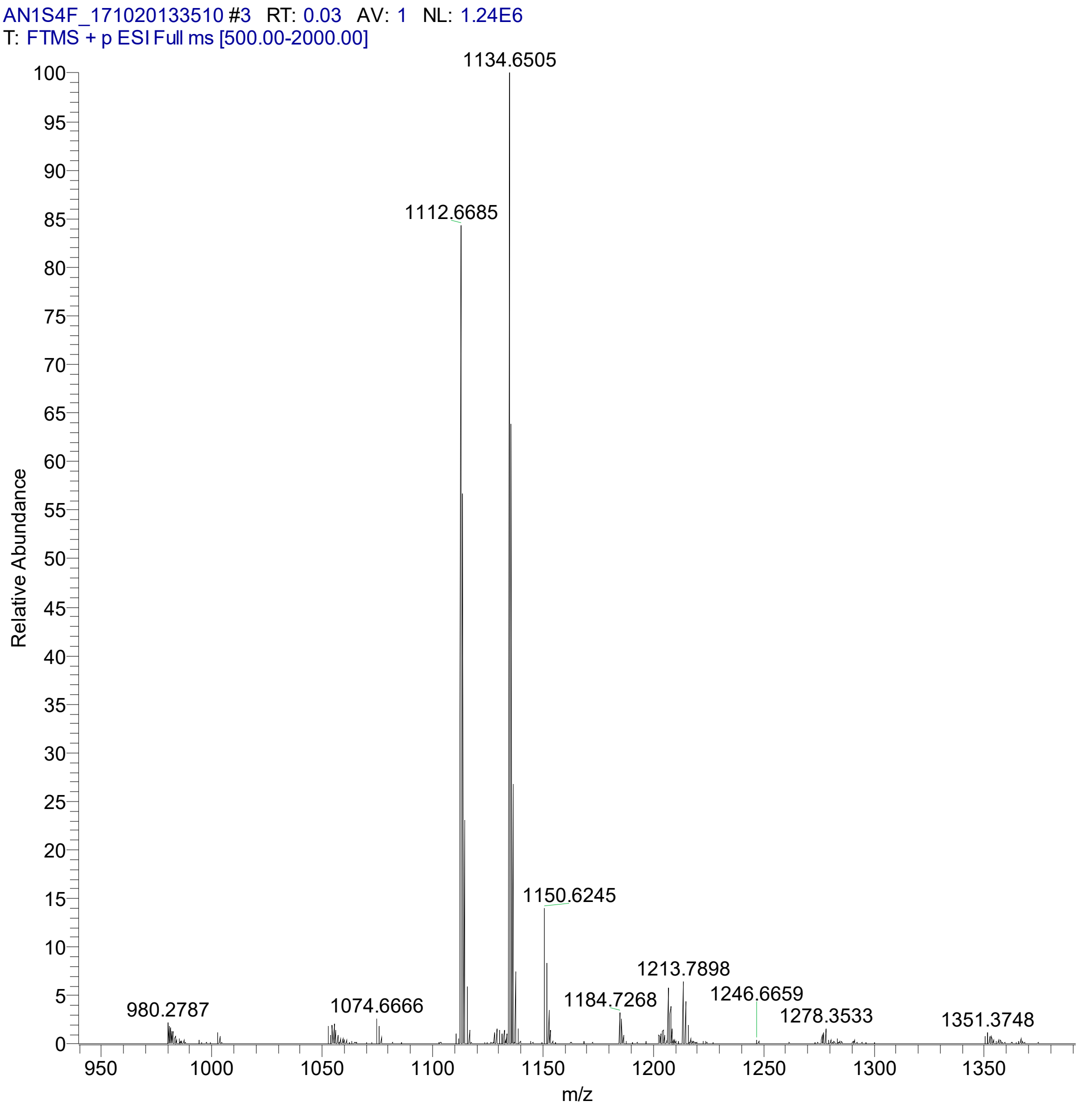

Figure S12. HRMS spectra of compound **C4**.

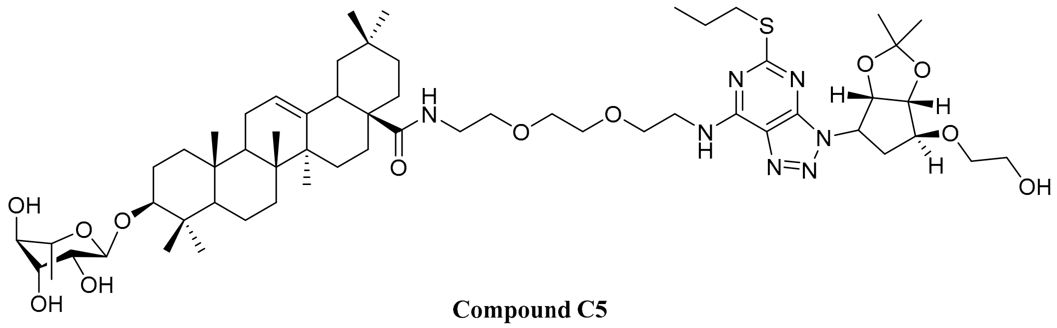

Compound C5

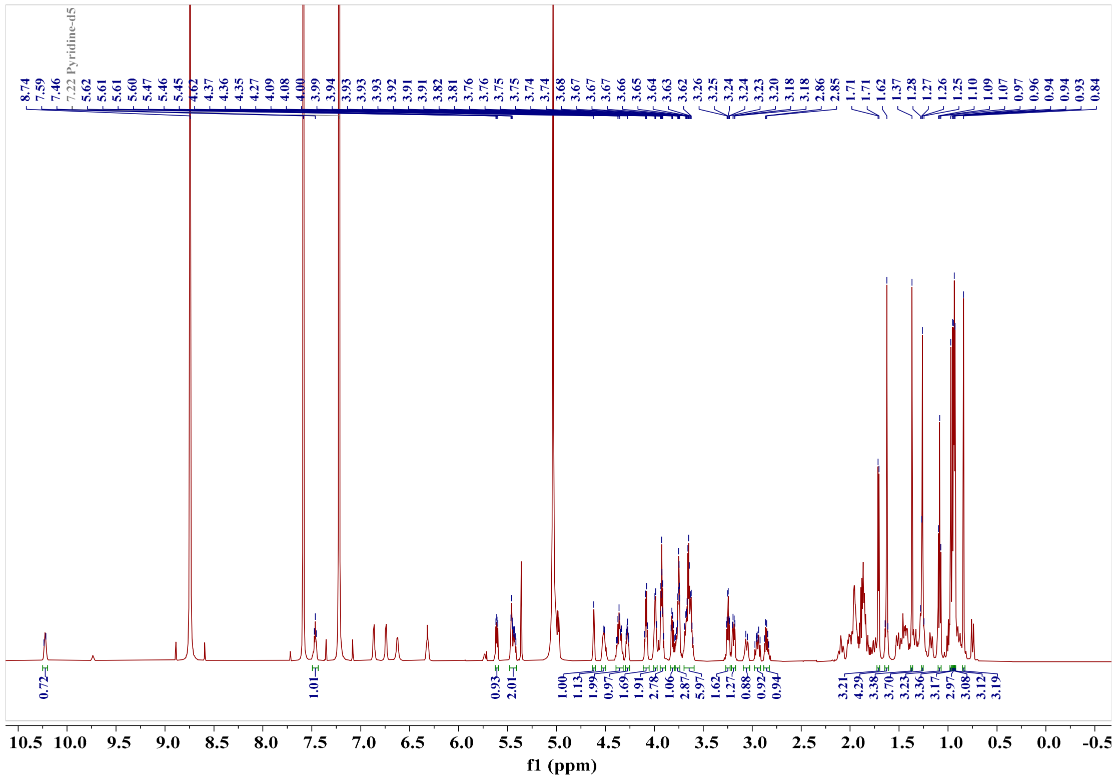

Figure S13. ^{1}H NMR spectra of compound **C5**.

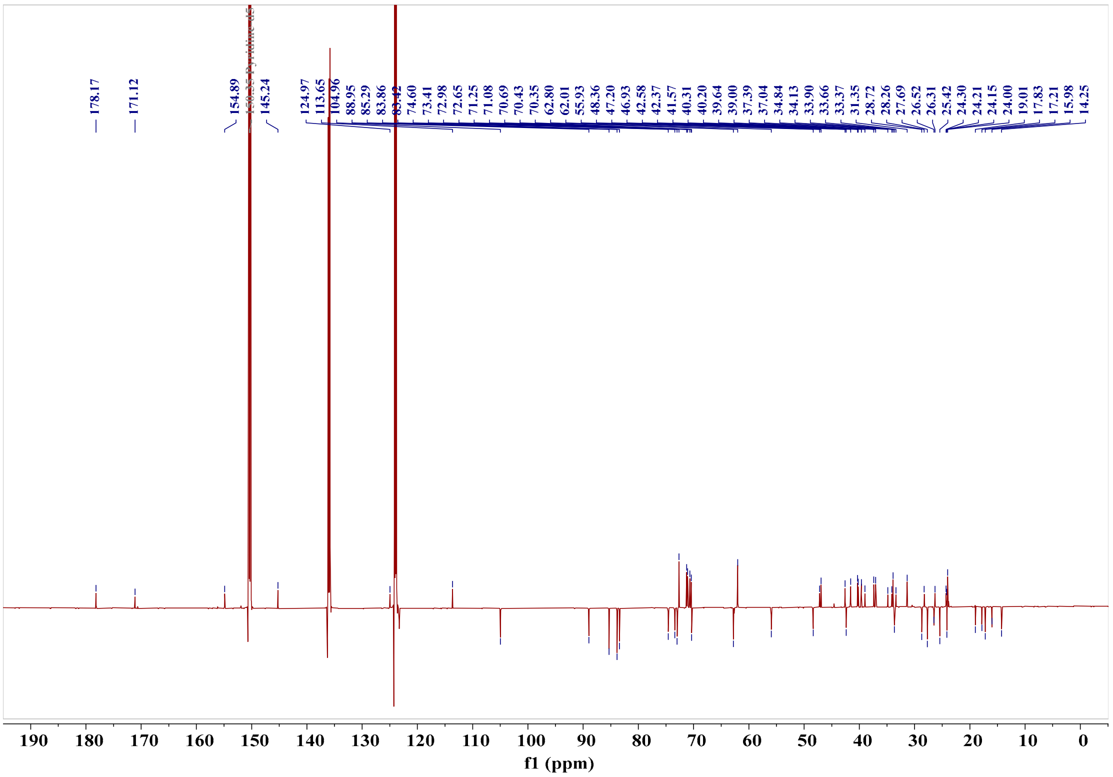

Figure S14. ^{13}C-NMR spectra of compound **C5**.

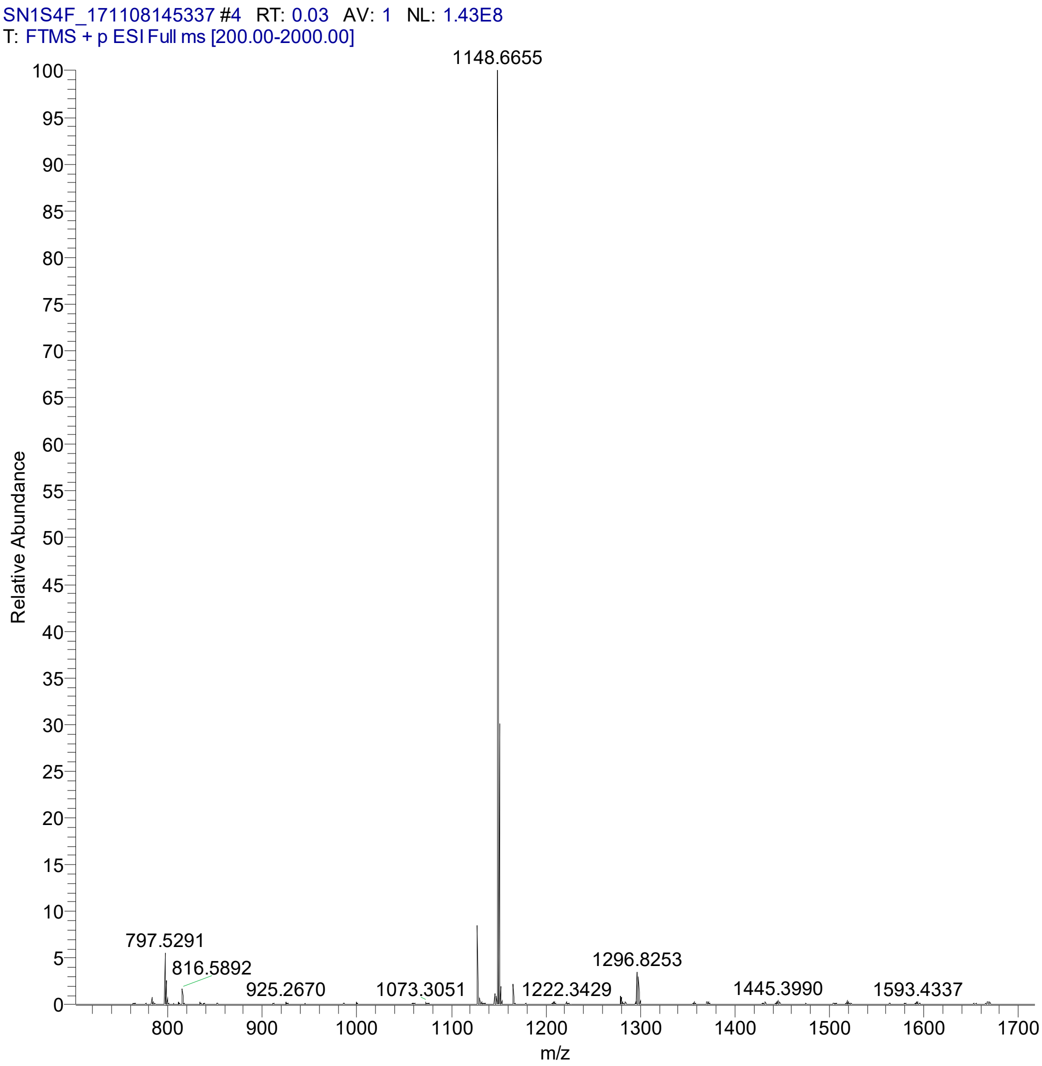

Figure S15. HRMS spectra of compound **C5**.

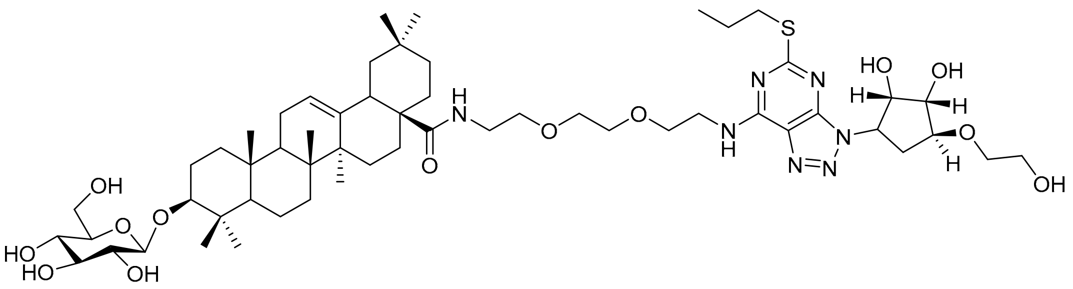

Compound C6

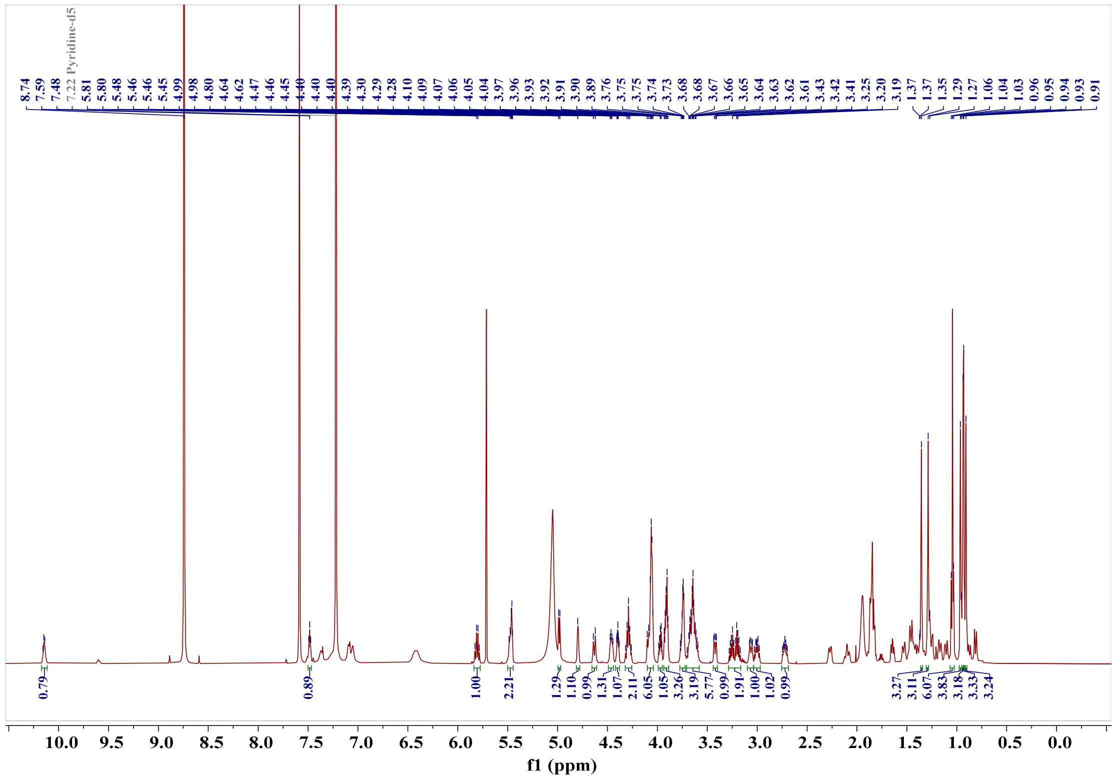

Figure S16. ^{1}H NMR spectra of compound **C6**.

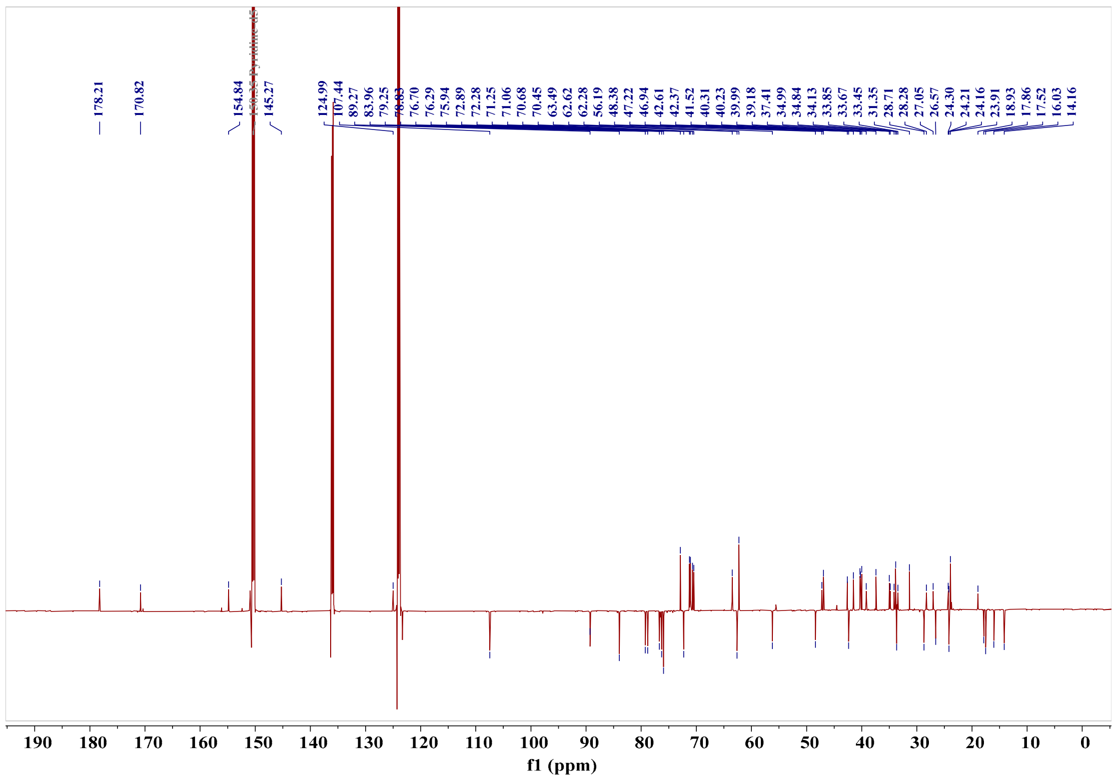

Figure S17. ^{13}C-NMR spectra of compound **C6**.

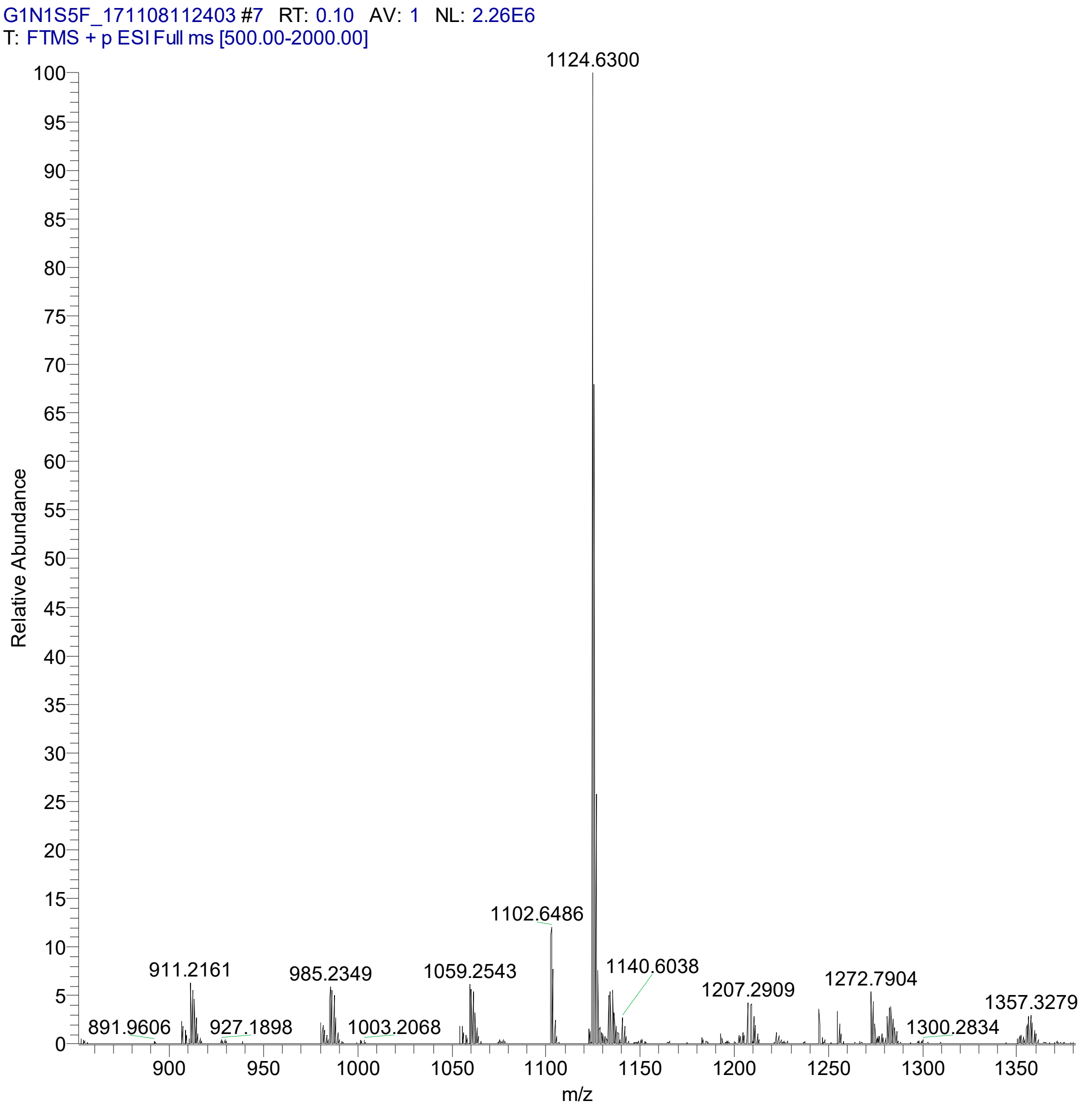

Figure S18. HRMS spectra of compound **C6**.

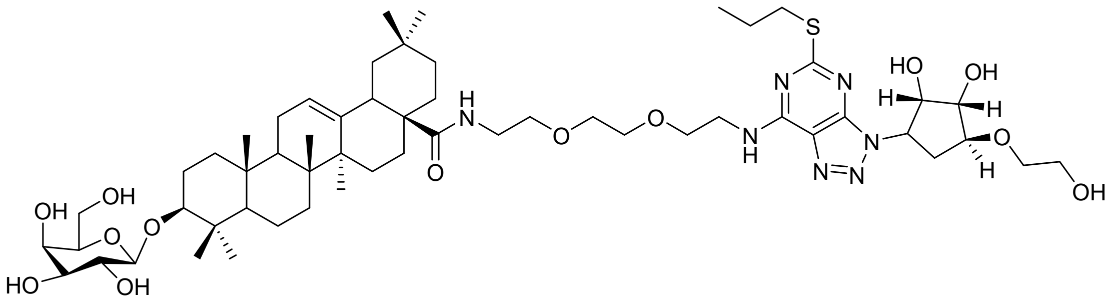

Compound C7

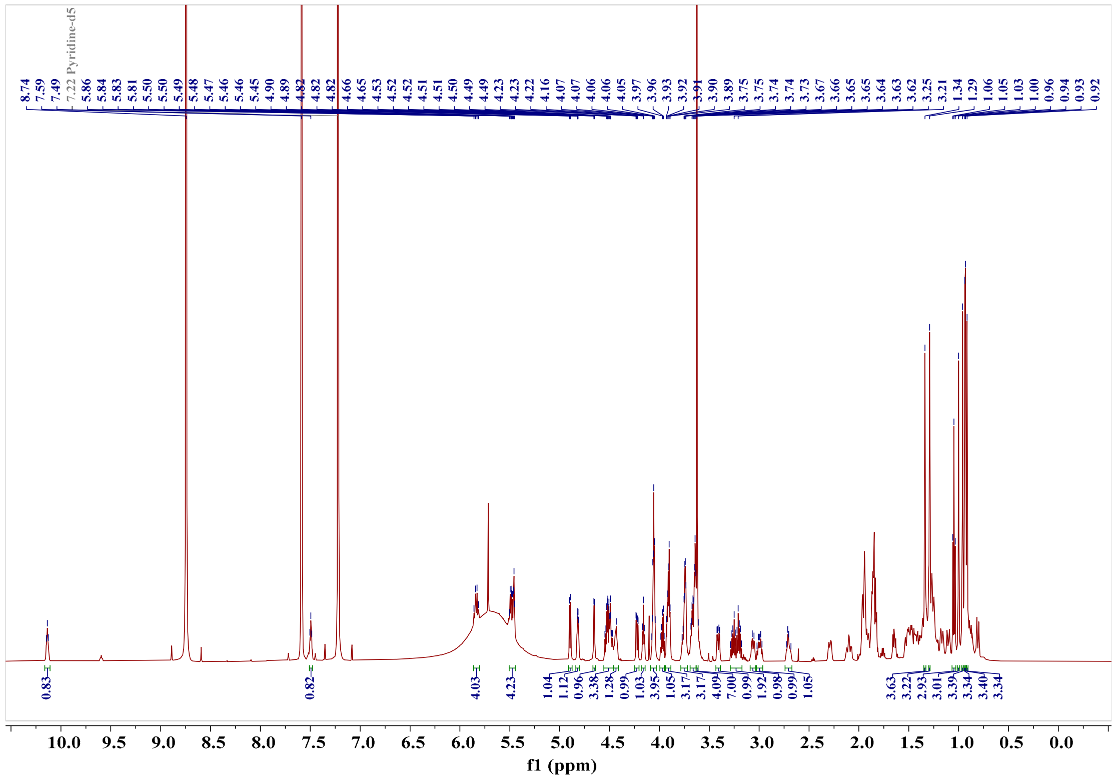

Figure S19. ^{1}H NMR spectra of compound **C7**.

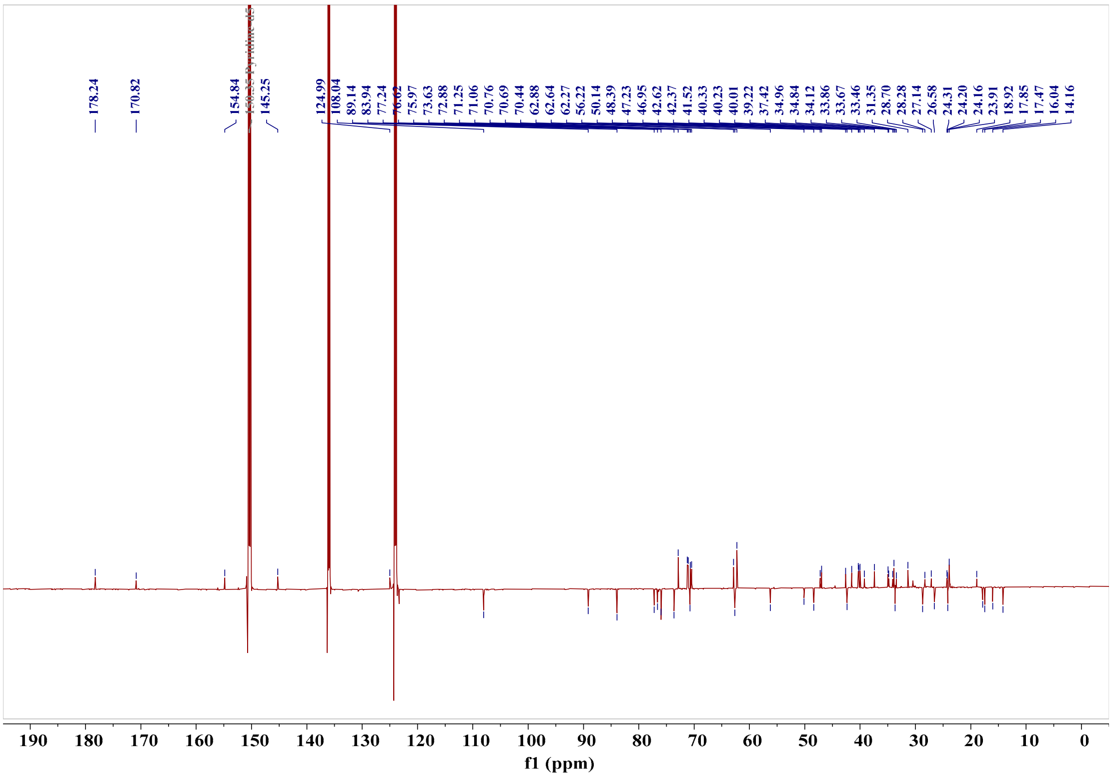

Figure S20. ^{13}C-NMR spectra of compound **C7**.

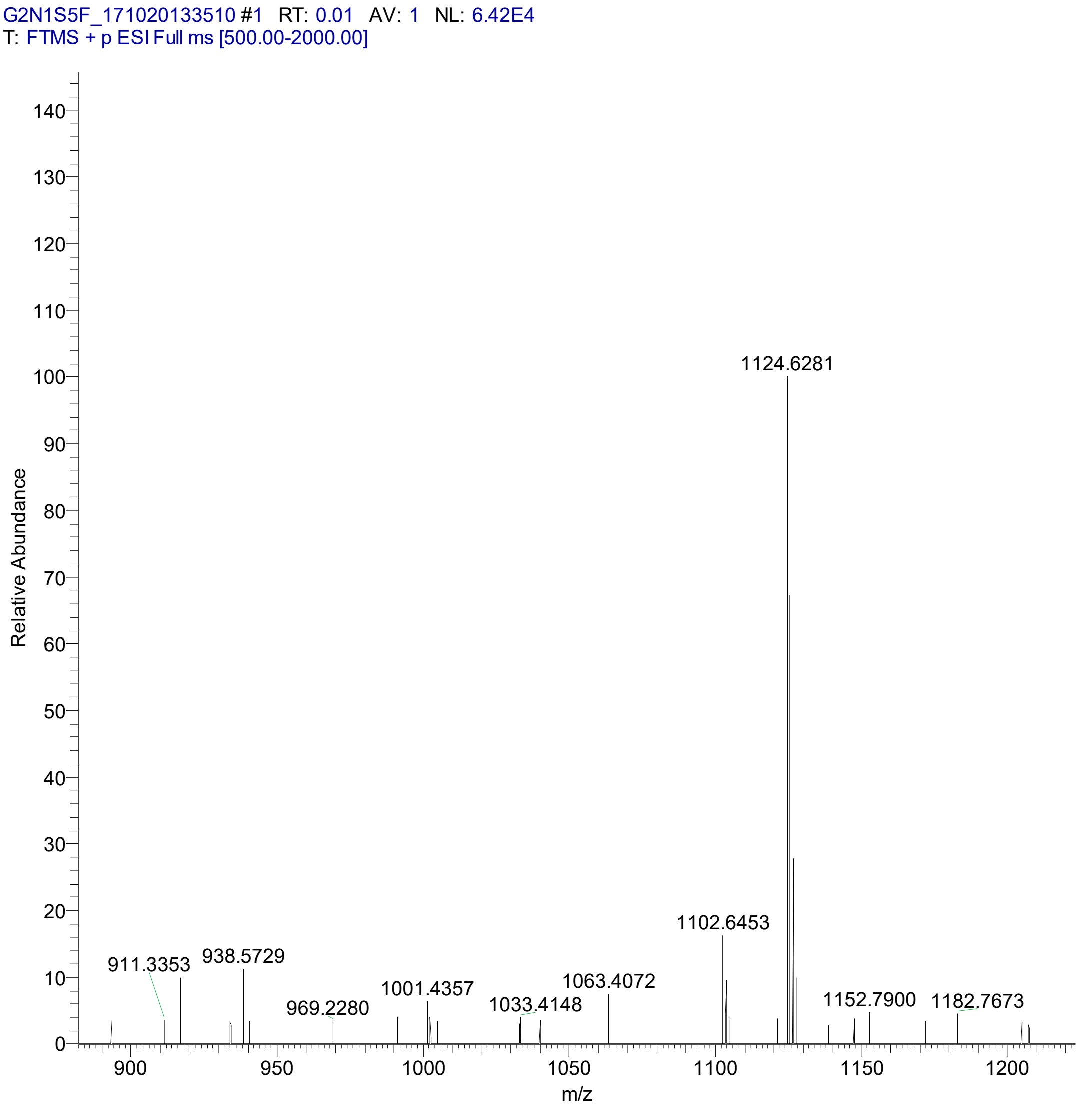

Figure S21. HRMS spectra of compound **C7**.

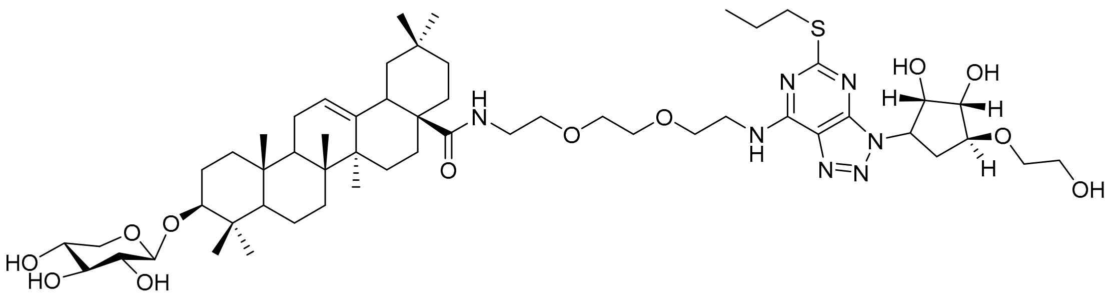

Compound C8

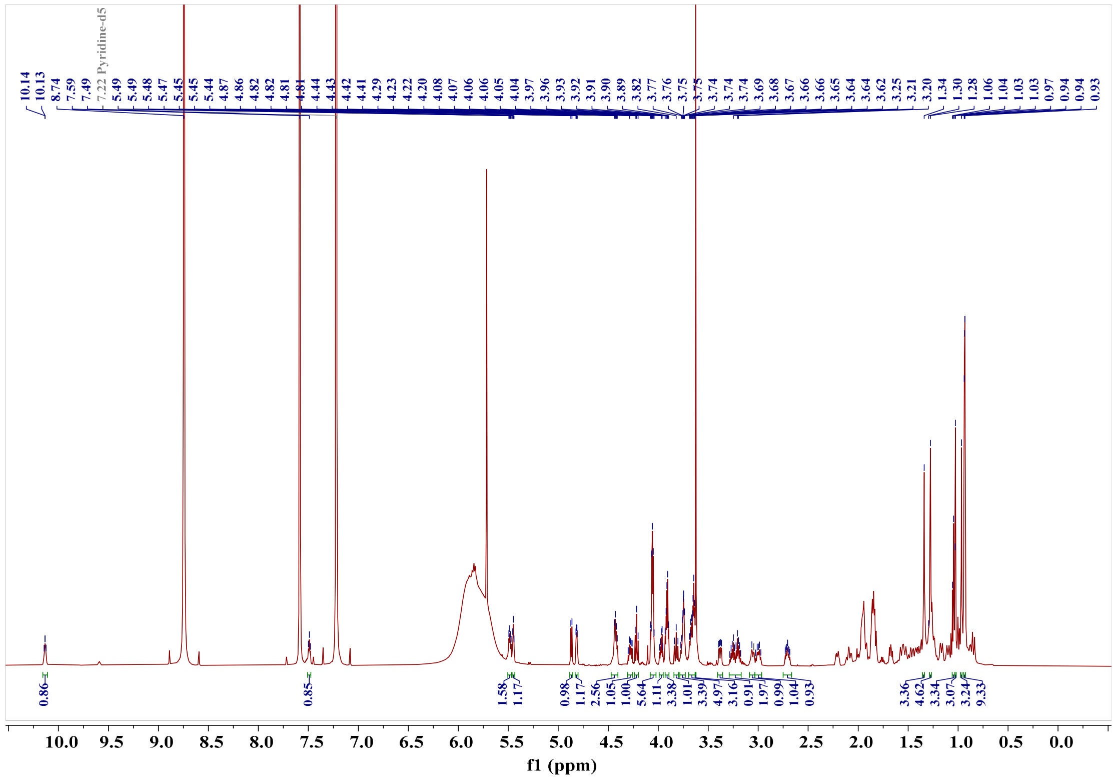

Figure S22. ^{1}H NMR spectra of compound **C8**.

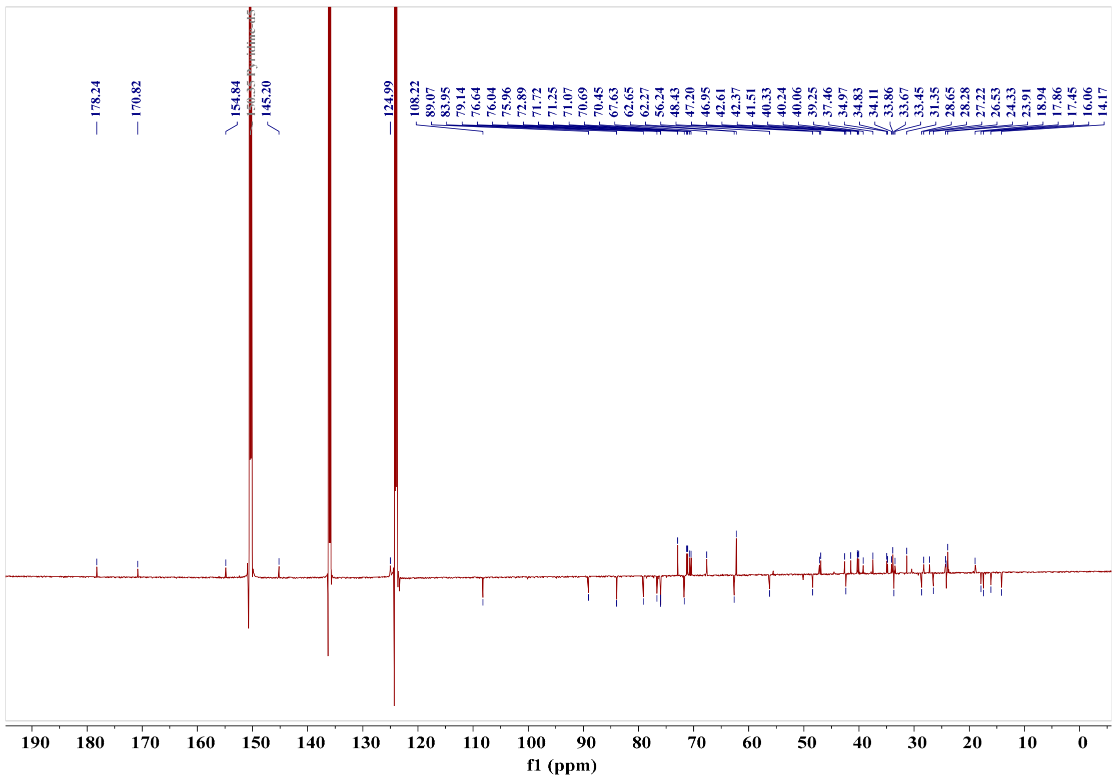

Figure S23. ^{13}C-NMR spectra of compound **C8**.

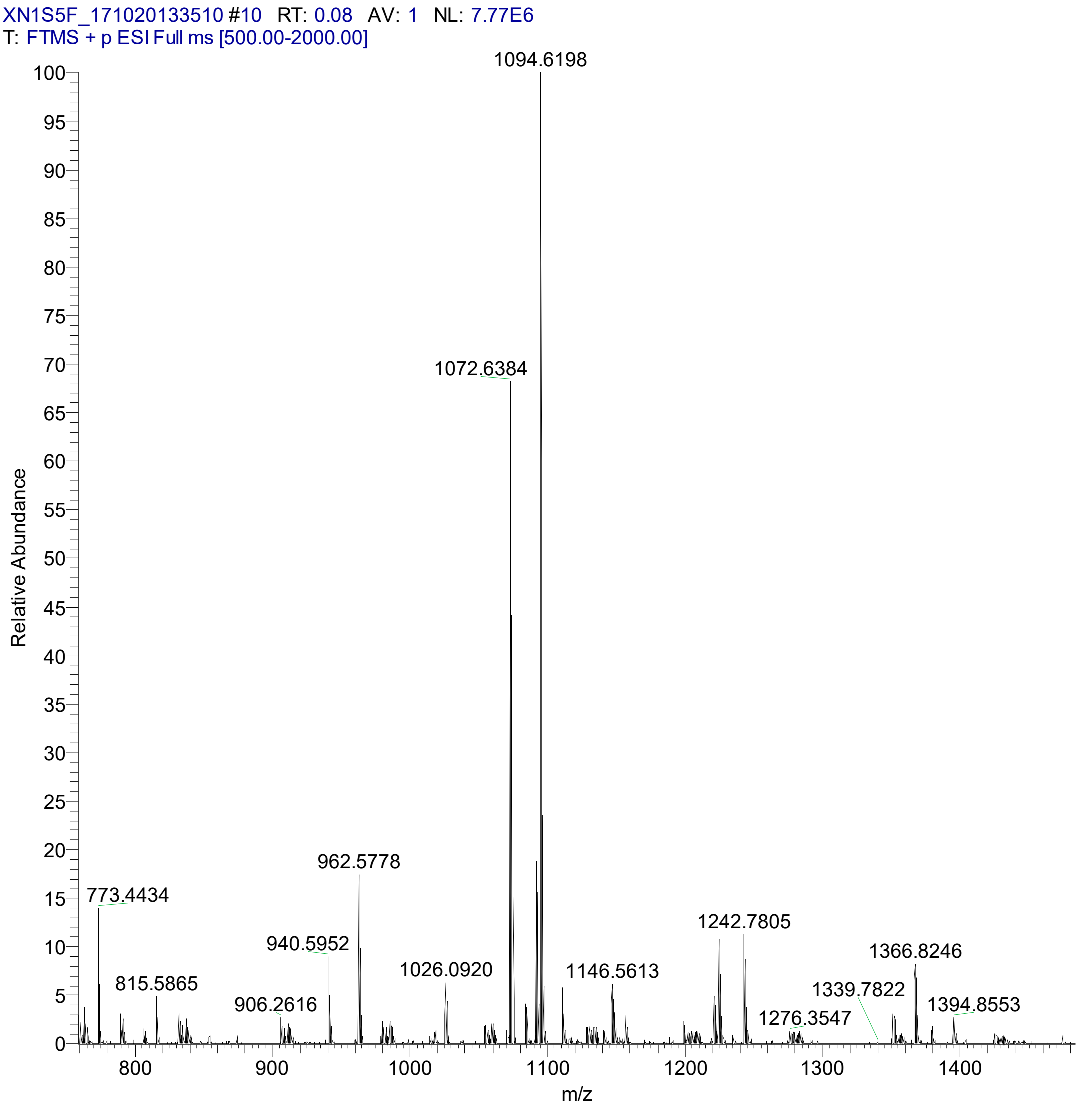

Figure S24. HRMS spectra of compound **C8**.

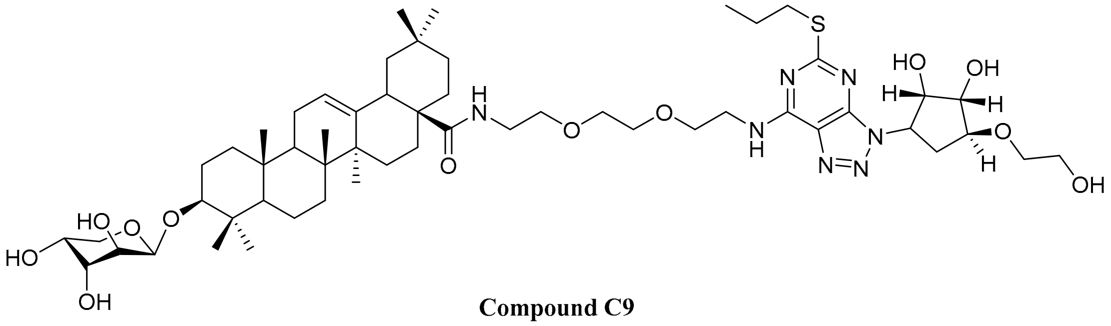

Compound C9

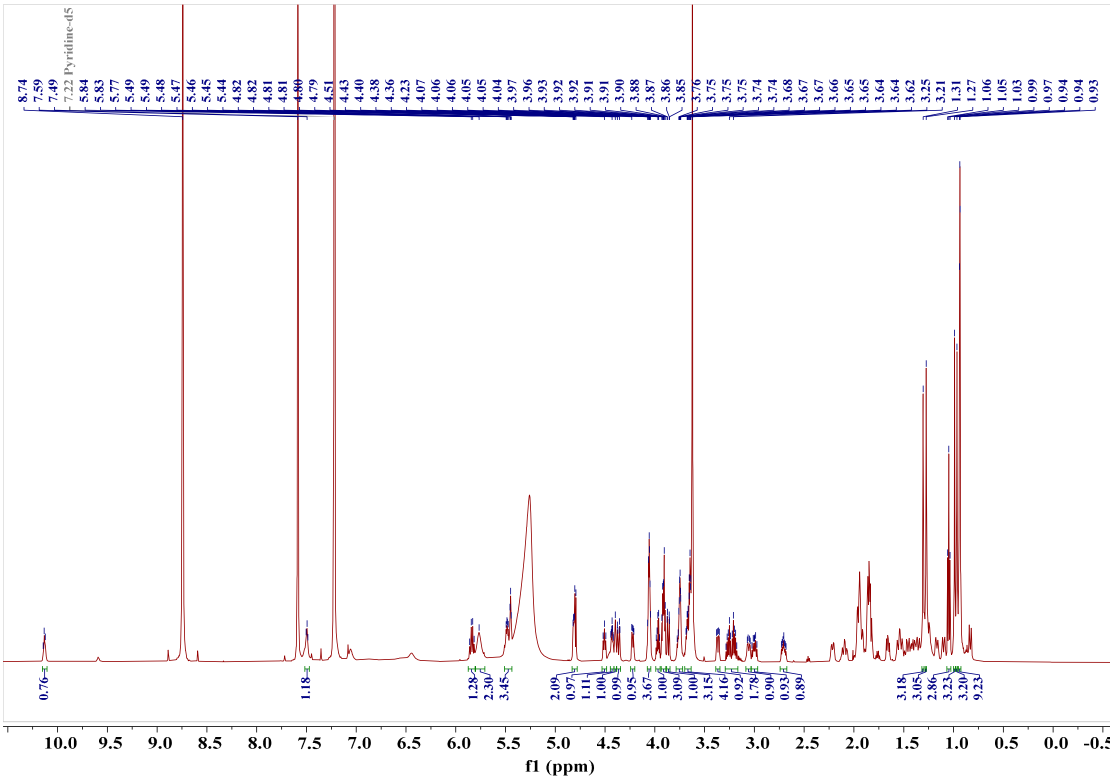

Figure S25. ^{1}H NMR spectra of compound **C9**.

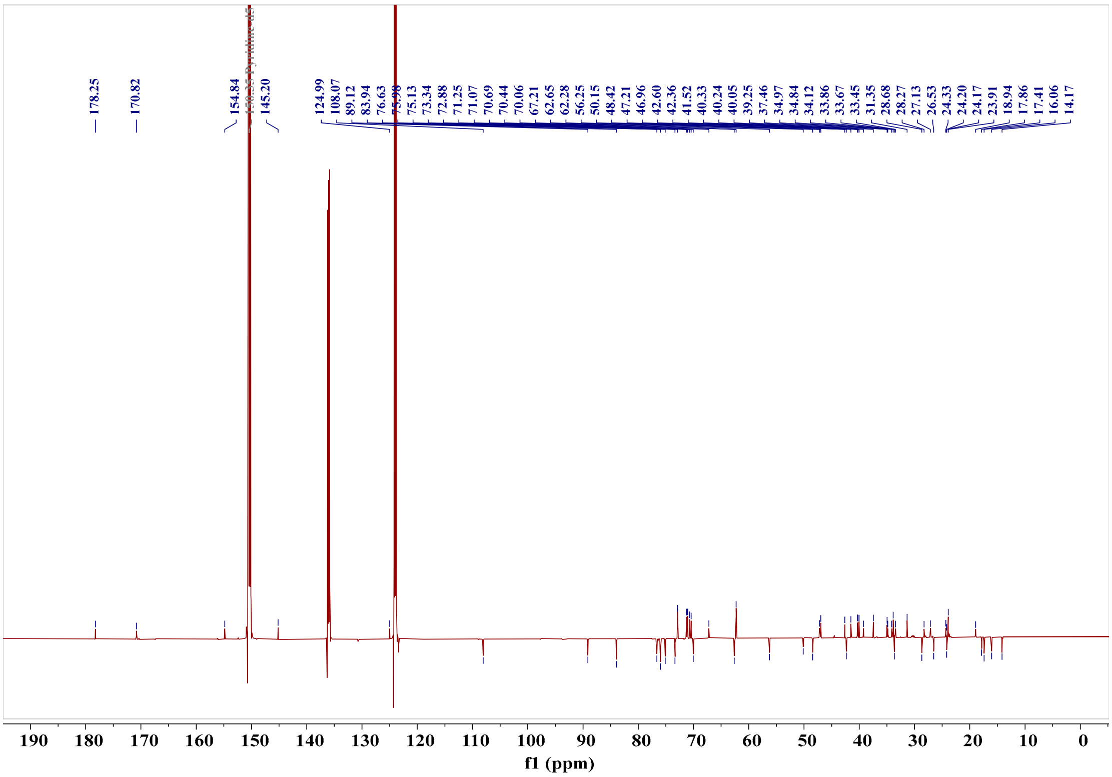

Figure S26. ^{13}C-NMR spectra of compound **C9**.

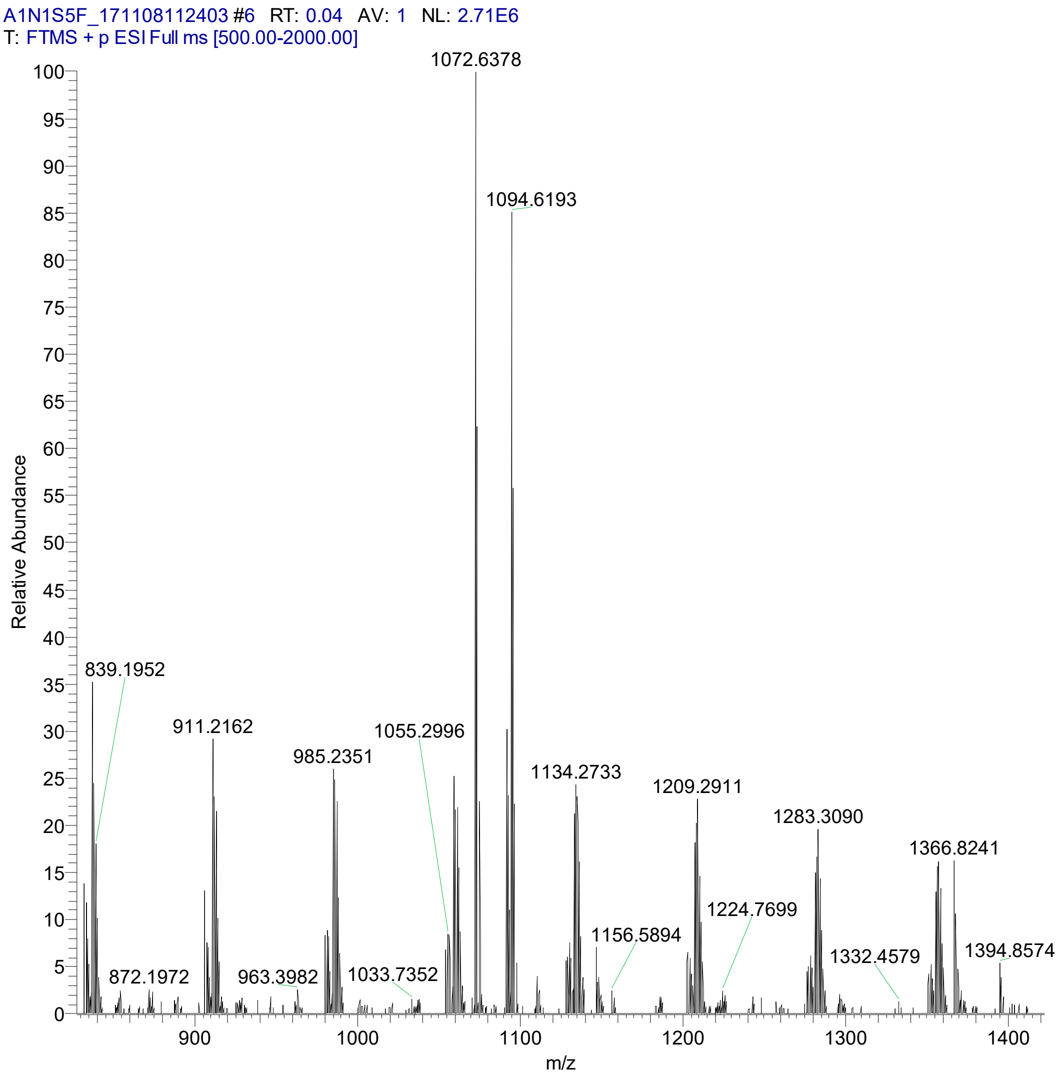

Figure S27. HRMS spectra of compound **C9**.

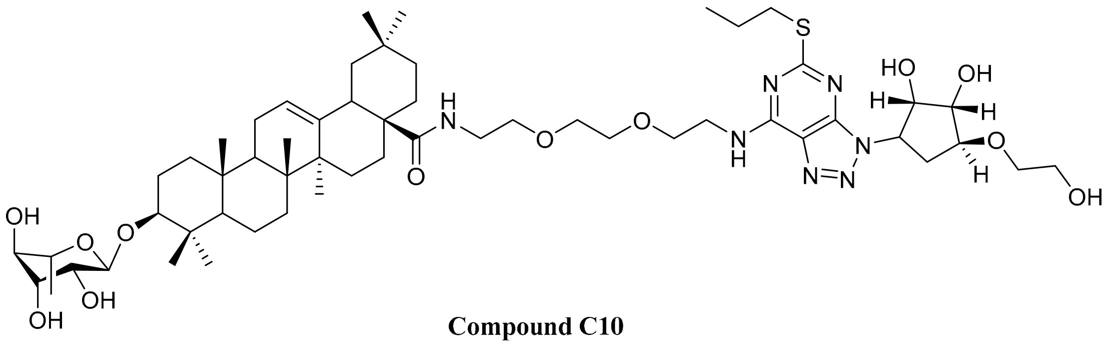

Compound C10

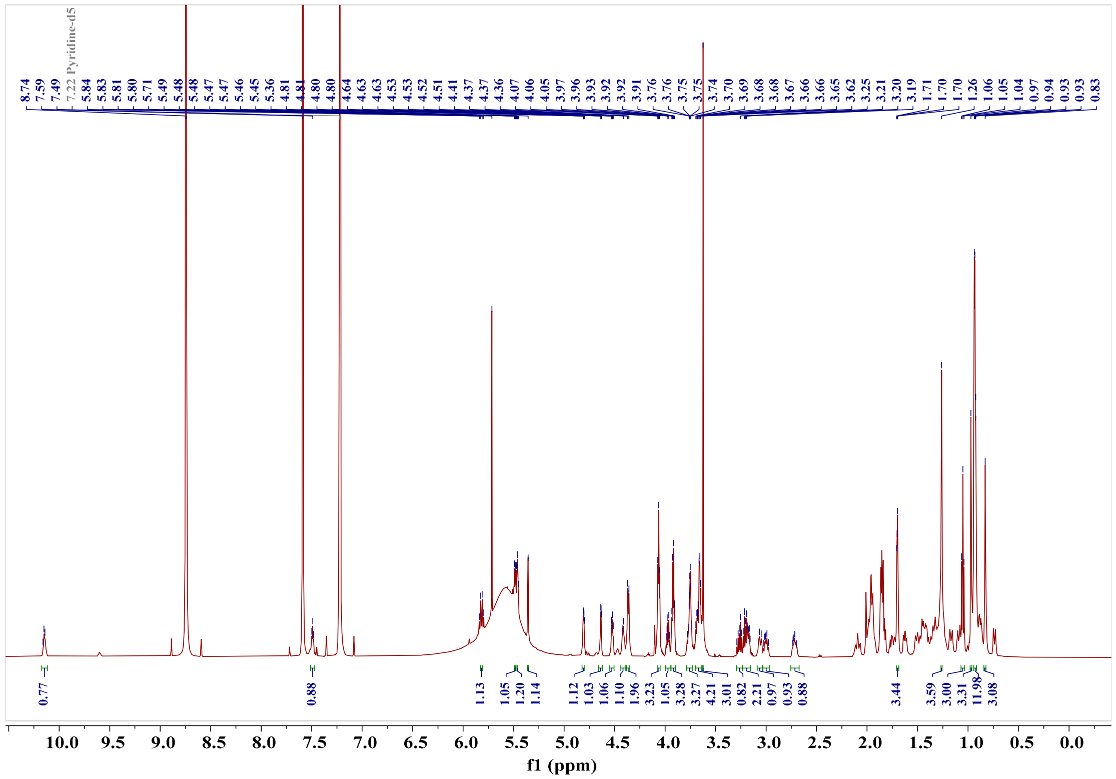

Figure S28. ^{1}H NMR spectra of compound **C10**.

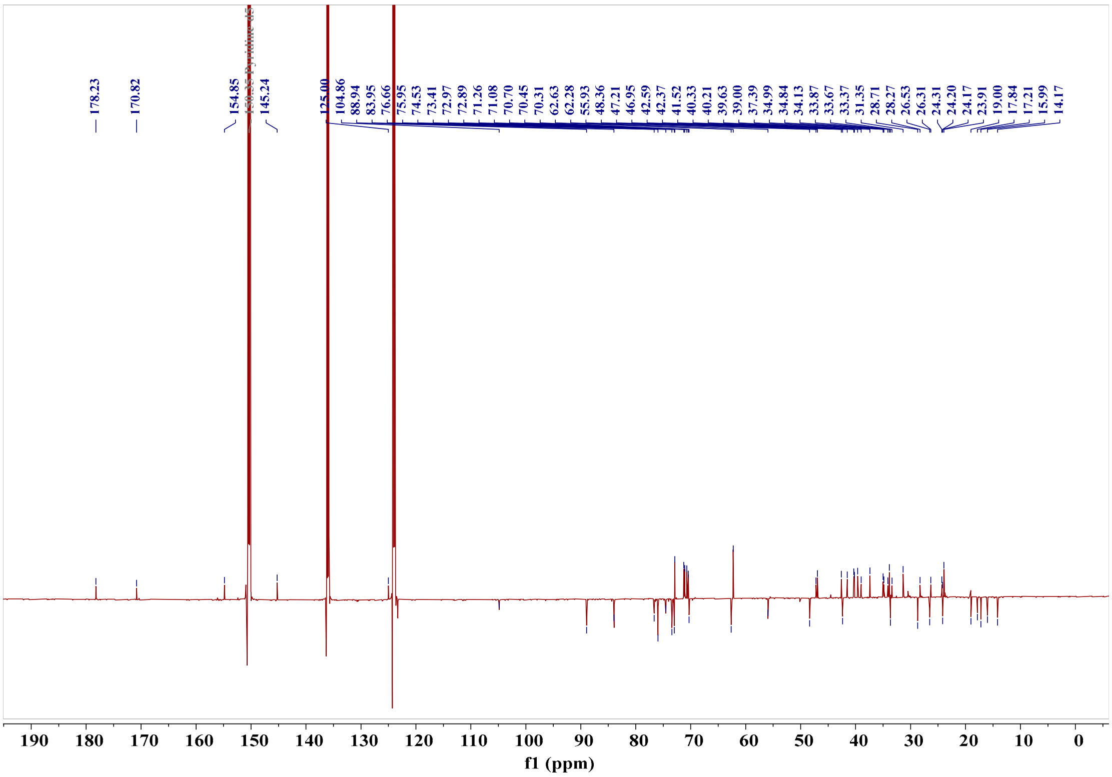

Figure S29. ^{13}C-NMR spectra of compound **C10**.

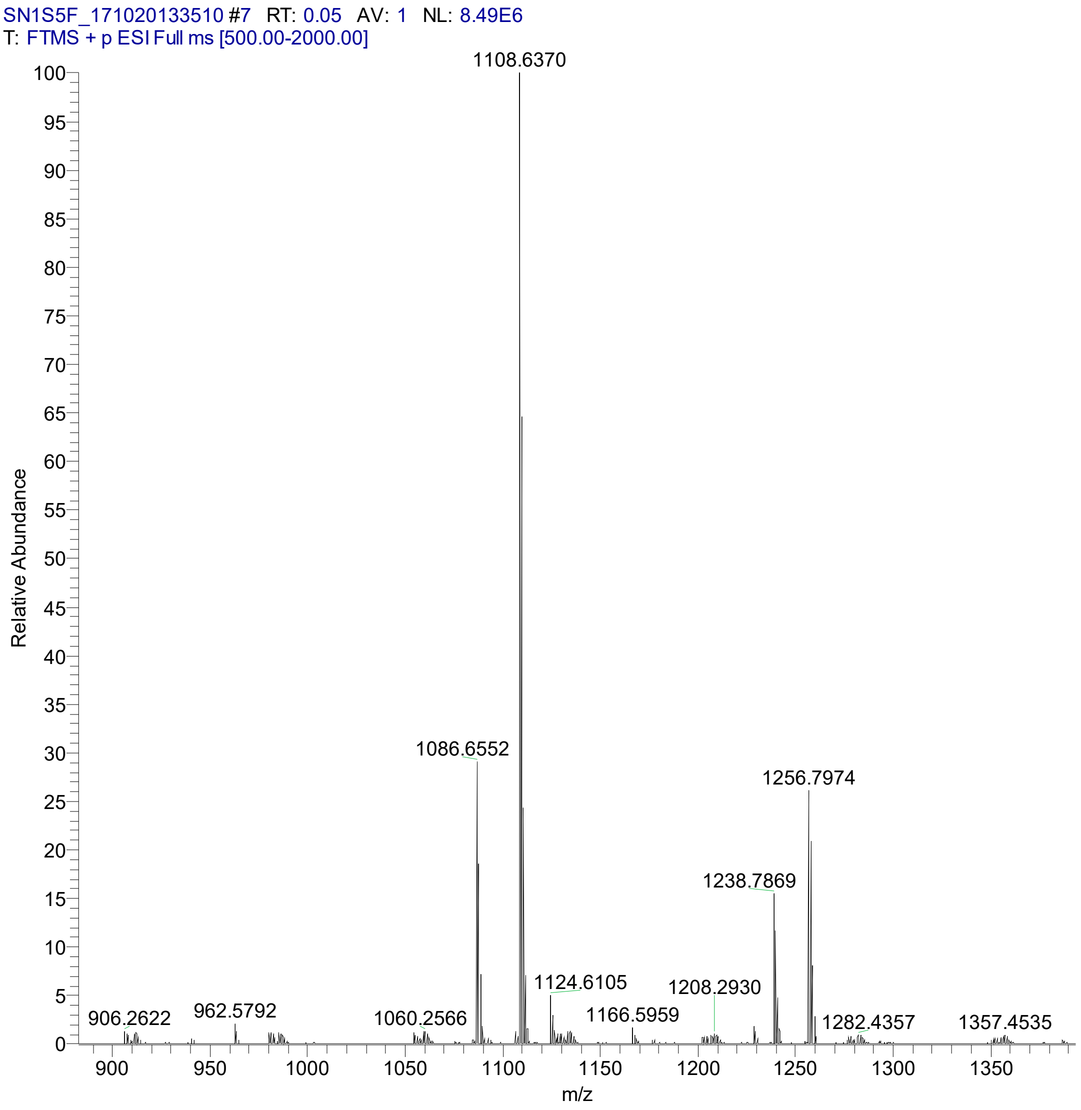

Figure S30. HRMS spectra of compound **C10**.

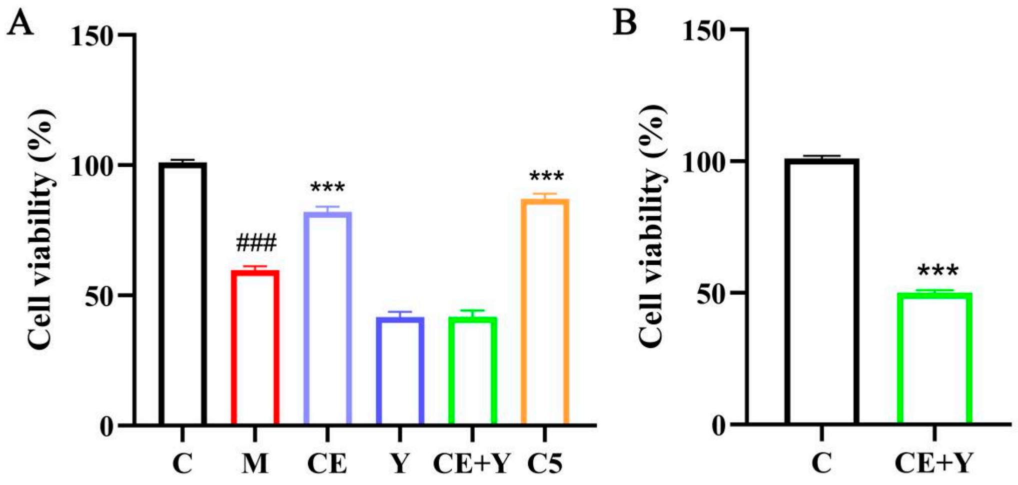

Figure S31. Protective effects of compound C5 against ox-LDL-induced damage in HUVECs cells. (A) Protective effects of compound C5, CE, ticagrelor and mixture

of CE and ticagrelor (1:1) at 3.125 μM. Data are presented as mean ± SD of each group (n=3). $^{###}P < 0.001$ vs Control group, $^{***}P < 0.001$ vs Model group. (B) The toxicity assessment of mixture of CE and ticagrelor (1:1) at 3.125 μM. Data are presented as mean ± SD of each group (n=3). $^{***}P < 0.001$ vs Control group.
